# Characterization of the APETALA2/Ethylene-responsive factor (AP2/ERF) transcription factor family in sunflower

**DOI:** 10.1038/s41598-018-29526-z

**Published:** 2018-08-01

**Authors:** Somayeh Najafi, Karim Sorkheh, Fatemeh Nasernakhaei

**Affiliations:** Department of Agronomy and Plant Breeding, Faculty of Agriculture, Shahid Chamran University of Ahvaz, P. O. Box 61355/144 Ahvaz, Iran

## Abstract

One of the most prominent families of genes in plants is the AP2/ERF which play an important role in regulating plant growth and responses to various stresses. In this research, a genome-wide survey was conducted to recognize the AP2/ERF genes in sunflower (*Helianthus annuus* L.), and a total of 288 *HaAP2/ERF* was obtained. Phylogenetic analysis divided them into four sub-families, including 248 ERF, 4 RAV and 35 AP2, and one subgroup of the Soloist family. Localization of chromosome, gene structure, the conserved motif, gene ontology, interaction networks, homology modeling, the modeling of *cis*-regulatory elements and the analysis of events in the duplication of genes were carried out for *HaAP2/ERF* genes. Finally, 9AP2/ERF genes were chosen to confirm the gene expression of the selected genes in leaf and root tissues in various abiotic stress conditions by qPCR. The results confirmed that AP2/ERFs genes could effectively resist abiotic stress. Also, proline content was studied under drought, salinity, cold and heat stress. The results indicated that proline was increased under abiotic stress. This research has been done for the first time to determine the *HaAP2/ERF* family, which prepared valuable data for the evolutionary and practical research regarding AP2/ERF in sunflower.

## Introduction

Plants express a number of species and numerous cultivars, genotypes, accessions etc. occurring in most parts of the world. Such resources are accepted as one of the most important plant genetic resources of biodiversity and support life system on earth. They are also important for human nutrition and health^1–4^.

Environmental stress causes physiological barriers for plants. In response to unfavorable environmental conditions, plants plan their cell rehearsal exercises through genetic monitoring systems such as post-transcription gene expression control. Transcription factors (TFs) and ncRNAs are two vital elements in functional genomics^5–7^. Development, progress and profitability of plants are irregularly affected by various biological stresses such as drought, salt, cold and heat. In order to survive and grow under abiotic stress, plants have created a complex reaction mechanism that prevents the development of genes with a different performance. As the main class of regulatory proteins, TFs assume the focal areas in the control system and identify the pathways for plant development due to biological stresses. Among the TFs, AP2/ERF is one of the largest plant TF super families which is likely to reduce the reactive ability of the highly patentable ethylene couple and the variable margins that contain 50–60 amino acids^8,9^. Due to similarities in the sequence and obstruction of the limiting region of AP2 DNA, it can be ordered to AP2, ERF and RAV^10^ families. While the AP2 family of proteins contains 2 AP2/ERF regions and is most often divided into the monophyletic group of AP2 and AINTEGUMENTA (ANT)^11,12^, the ERF subgroups have a specific WLG domain and it’s divided into 10 groups^10^, where Groups I to IV have a subgroup of DREB, and Groups V to X have the subgroup of ERF. ERF is represented by an additional *Cis*-acting component of the AGCCCGCC of the GCC-enclose in the region of the promoter^13^, while the DREB sub-region usually responds to the drying of the constructor limiting variable having the CCGAC central theme^14^. The relatives of the RAV contain the single region of AP2/ERF and a particular B3 DNA-binding motif^15^. Additionally, different accessions with Ap2-like domain but due to the extra motif are regularly identified as Soloist^10^.

Extensive examinations have deciphered the essential part of AP2/ERF genes in the growth of plant, improvement and stress response^11,16–18^. For the most cases, the AP2 subfamily the main elements of organic design and organic progression, for example, the determination of epidermal leaf cells, spikelet meristem and the design of plant organs^19^ and grain yield^20,21^, while the RAV subfamily demonstrated significant operations in transduction of plant hormone, including ethylene^22^, brassinosteroids^23^, and responses to biotic/abiotic stress^24,25^. In addition, the DREB, along with a different individual in ERF family, is mainly affected by biological and abiotic stresses, for example, water defect^26^, low temperature^27,28^ and high salt stress^29^. Proline free accumulation is a common reaction to stress in high plants^30^. There are several reports of positive correlations between proline accumulation and the compatibility of plants with stress conditions under drought stress and salinity^31^. Proline affects the solubility of various proteins and enzymes and prevents them from changing their nature. In plants such as beans and soybeans, there has been a significant increase in proline content as a result of a decrease in water potential^32^.

As a major oil seed crop, sunflower (*Helianthus annuus* L.) is impervious to different abiotic stress because of its different forms of metabolism, physiology, and methods of regulating the reproductive stress metabolism. This function is of unique enthusiasm for adjusting it to high temperatures, limited access to water, high salinity and predominant metal scrap in soil^33^. Typically, the DREB subfamily as a candidate can possibly increase the environmental tolerance of the product. The DREB subfamily shows distinct reaction patterns relative to ecological strategies including low temperature (*AtCBF1*)^27^, heat (*ZmDREB2A*, *AtDREB1A*)^28,34^, osmosis (*CkDREB*)^35^, drought (*OsDREB1*)^26,36^ and the lack of water and high stress (*CaDREBLP1*)^29^. DREB provide a large number of hydration/cold genes in collaboration with the DRE/CRT components (A/GCCGAC) available in COR/RD promoters^37^. However, a few genes from the DREB subfamily have been reported to be positive and negative intermediary of ABA and sugar reactions. This is especially true during both germination and the initial stages of plant breeding^38^.

Over-expression of the DREB gene within the framework of plants increased salt tolerance as a positive control^39,40^. Expression of *OsDREB2A* and *OsDREB1F* enhancement increased drought/salinity stress in rice and Arabidopsis^40^. In rice, cold stress created *OsDREB1A* and *OsDREB1F*. *OsDREB1F* was also used for drought, salt, and ABA treatments. Over-expression of *OsDREB1A* and *OsDREB1F* led to increased resistance to dry season and severe salt susceptibility in Arabidopsis^39–41^. A reverse genetic approach could identify a CBF2 mutation in Arabidopsis in which the CBF2/DREB1C gene was abnormal. The mutation of CBF2 had increased resistance to drought stress and salt. The expression analysis showed the inconsistency of CBF2/DREB1C with CBF1/DREB1B and CBF3/DREB1A instructions^42^. Surprisingly, the DREB1/2 suggesting there was a cross-talk between them under drought and salt stress^41,43–46^. These results indicate that the DREB1 and DREB2 gene elements, when combined with ABA, are not only maintained in monocotyledonous and dicotyledonous plants but also play important roles in drought and salt stress conditions^39^.

Typically, genome information encourages the recognition of gene function and increases the definitive information required to comprehend the molecular component of stress reactions, thus increasing the abiotic tolerance of various products to date. The AP2/ERF family is known in Arabidopsis^8^, bamboo^47^, grapevine^48^, maize^49^, peach^50^, and rice^51^. To the best of our knowledge, no systematic proof of the AP2/ERF family has been accomplished in sunflower. Moreover, the limitations of research on its genetic family are essential.

In this study, a broad bioinformatic research led to the discovery of genomic linkage, the phylogenetic relationship and the gene expression of AP2/ERF genes in *Helianthus annuus*. Also, the researchers analyzed chromosome localization, gene structure, gene ontology, homologous modeling of *HaAP2/ERF* protein, *cis* components in the promoter region, gene amplification, and evolutionary systems. Our research is aimed at grounding the way for adding to the families of AP2/ERF family control in the advancement of sunflower in response to abiotic and biotic stress, which not only does provide supportive data on changing the mechanism of evolution of this TF family in the plant but it also adds to the discovery of a molecular system for the improvement of stress response in the above mentioned plant and other crops of different species.

## Results

### Reconnaissance of AP2/ERF family in sunflower

Overall, 288 genes were recognized as acceptable AP2/ERF genes in sunflower. The predicted *HaAP2/ERF* genes (generic name and locus Tag has been shown in Supplementary Table S in details) were then chosen according to the location of chromosome and their family classification (Table S1). Based on the classification, we categorized them into21 ANT, 14 AP2, 4 RAV, 1 Soloist, 105 DREB, and 143ERF. Thirty five AP2 TFs genes including2 AP2 domains or a single AP2 domain which were similar to AP2 domains in the double domain groups were assigned to the AP2 family, and four genes together with the B3 type domain were classified into the RAV family.248 genes had only one domain belonging to the ERF subfamily, which was itself divided into ERF and DREB subgroups. In addition, a specific gene called *HaAP2/ERF-288* resembled other family members which were in the Soloist subgroup (Table S2).

**Figure 1:**
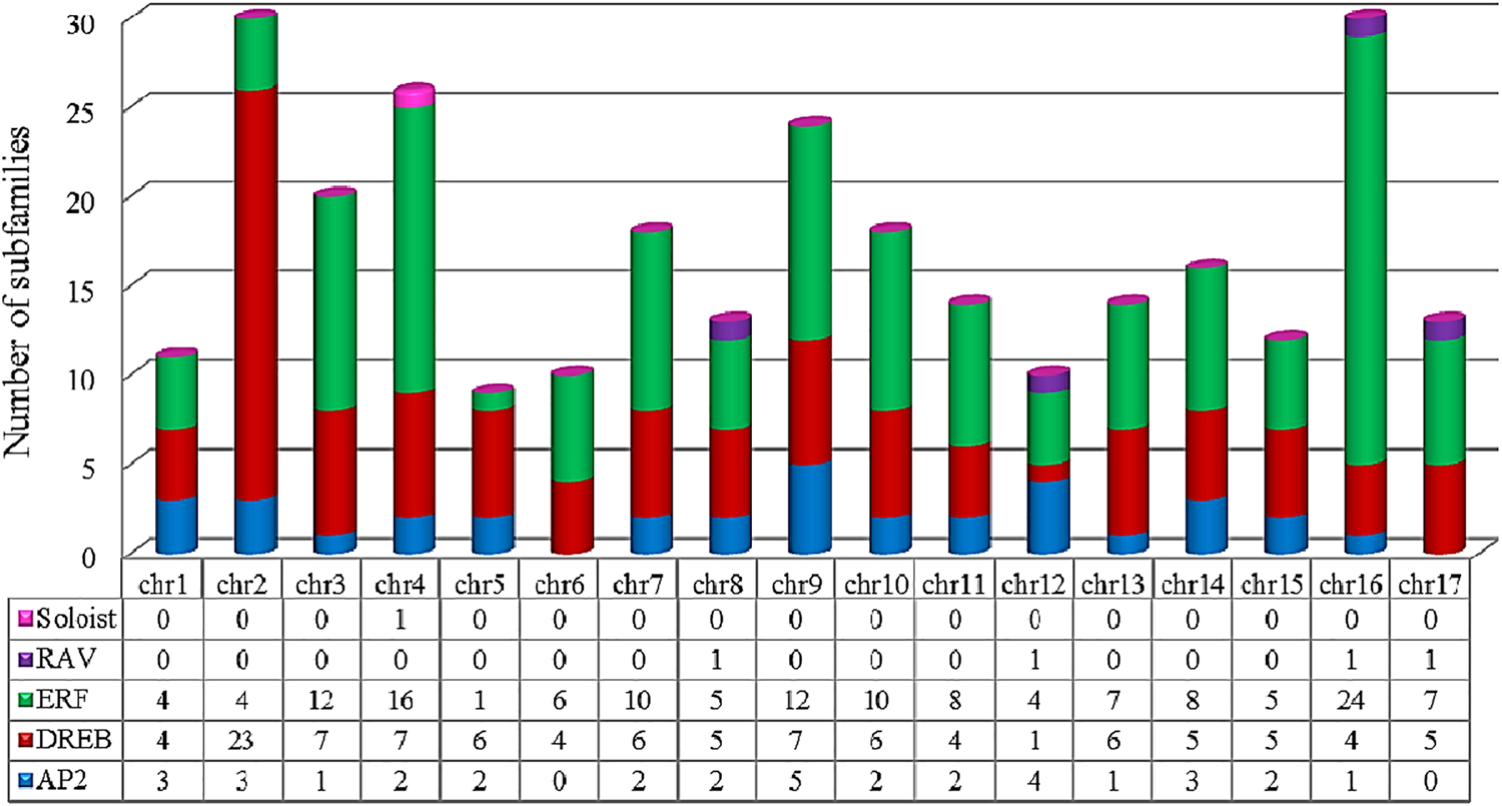
Chromosome-wise distribution of 288 AP2/ERF genes on 17 chromosomes of *Helianthus annuus*.

Distributions of *HaAP2/ERF* genes on 17 chromosomes of sunflower were unequally demonstrated (Fig. 1), with the maximum and minimum number of AP2/ERFs located on chromosomes 2 and 16 (30 genes) and chromosome 5 (9 genes), respectively. The length of specified proteins of *HaAP2/ERFs* ranged from 7 to 1347 amino acids. Their molecular weights (MW) ranged from 8.52 to 69.44 kDa and their theoretical isoelectric points (PI) ranged from 4.48 to10.27. Prediction of protein localization in cellular compartments revealed that the majority of *HaAP2/ERFs* (234 of 288, 81.25%) were located in the nucleus while 54 genes were located in the extracellular compartment (Table S1).

### Phylogenetic relationship, conserved motif and gene structure analysis

**Figure 2:**
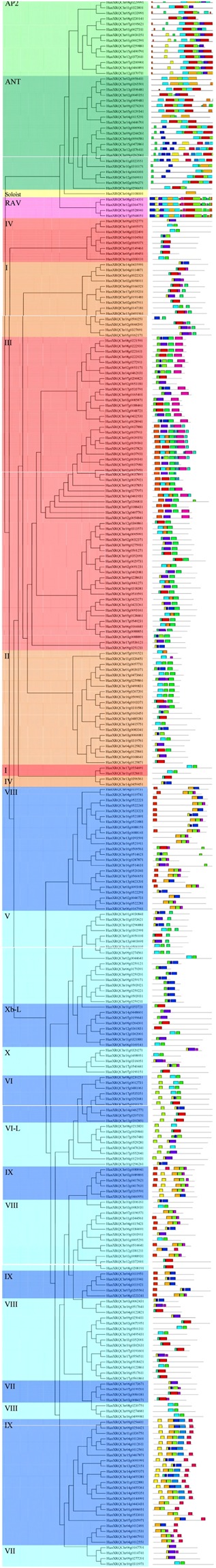
Conserved motifs analysis of *HaAP2/ERF* genes according to the phylogenetic relationship. Each motif is represented by a number in a colored box. Box length corresponds to motif length.

**Figure 3:**
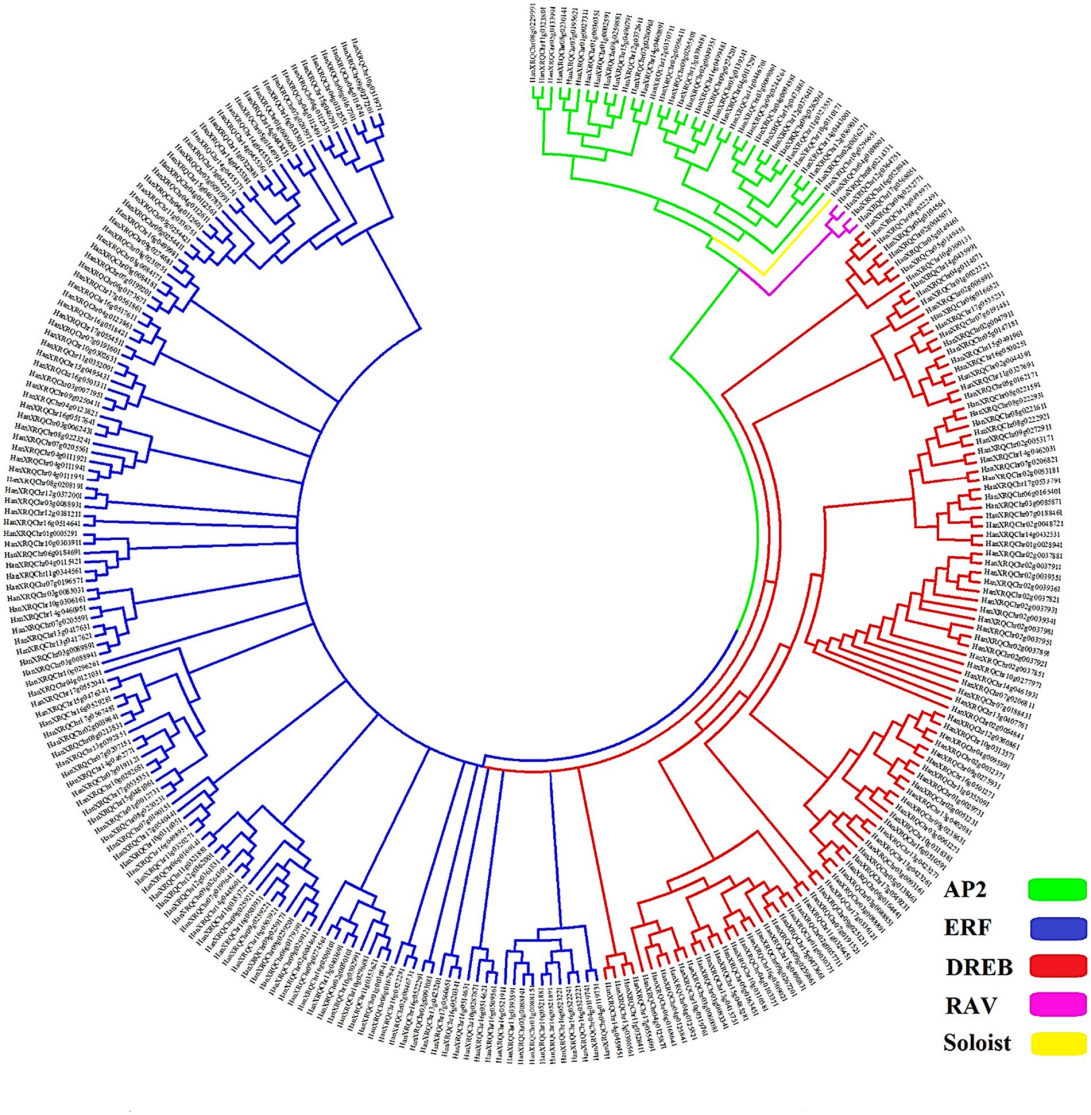
An unrooted phylogenetic tree of AP2/ERF family proteins in *Helianthus annuus*. The complete sequences of 288 AP2/ERF family proteins identified in this study were aligned by ClustalX2.1 and the phylogenetic tree was constructed using the neighbor-joining method with MEGA7.0 software.

**Figure 4:**
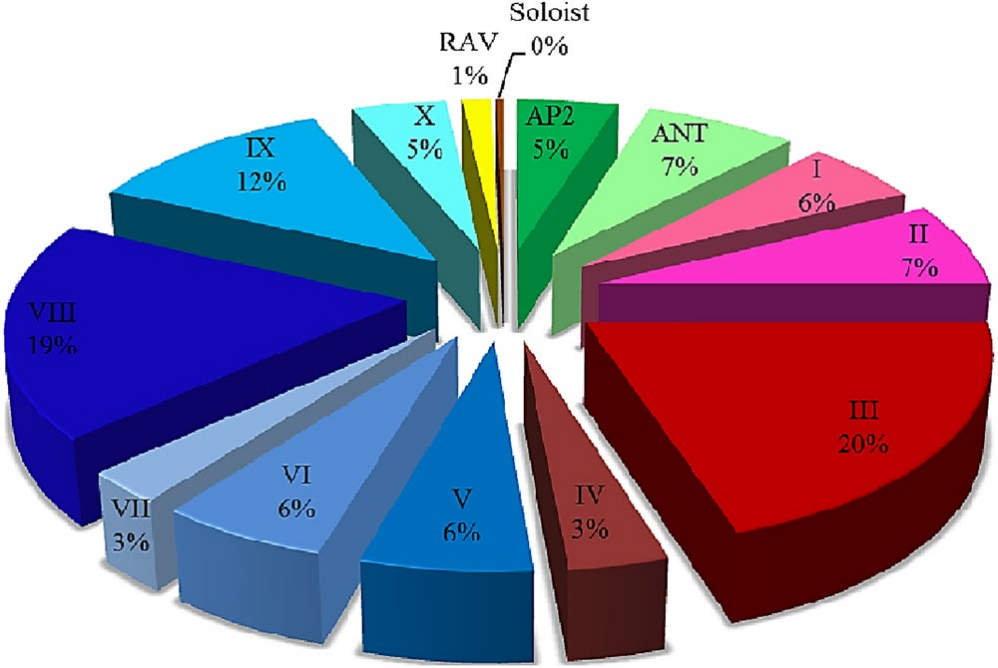
The percentage of genes belonging to different groups *HaAP2/ERF*.

To assess the evolutionary associations of the *HaAP2/ERF* genes, phylogenetic analysis was performed based on the multiple correlations of all *HaAP2/ERF* with ArabidopsisAP2/ERF genes. The NJ tree categorized the studied genes into three main categories of ERF, AP2, and RAV based on the composition of their amplitude as described in Fig. 2. A nunrooted phylogenetic tree was made with *HaAP2/ERF* family proteins (Fig. 3). Also, ERF clades were divided into 10 groups. Similar to Arabidopsis assortment criteria^6^, ERFs families could further be subdivided into a subgroup of DREB and the ERF families. Four groups (I–IV) belonged to DREB and the remaining 6 groups (V-X) were the ERF subgroups (Fig. 4). It was found that DREB subgroups contributed mainly to environmental reactions. Several of DREBs genes that inducible by stress from numerous plants have been recognized to date^28,29,52^. Identification of DREB genes of *Helianthus annuus* provides valuable resources for the characterization of stress-response genes. In addition, the bootstrap values of the nodes in the NJ tree were not very high in each class, which was in accordance with earlier studies^10,53^. The reliability of the NJ tree was confirmed by the production of another phylogenetic tree using the maximum parsimony analysis (MP). It was recognized that almost all *HaAP2/ERF* were located in similar clustering groups. In addition, *HaAP2/ERFs’* retained motifs were analyzed and considered. Altogether, twenty-five conserved motifs were identified (Fig. S1). Among them, 9 subjects included motifs 1, 2, 3, 4, 5, 7, 10, 11 and 12 in the AP2/ERF range, while 16 were linked to areas outside the DNA-binding domain. Seemingly, these are as contain either functional factors or are related to nuclear positioning and transcription regulation^54^.

### Conserved amino acids in *HaAP2/ERF* transcription factors family

Amino acid 30 G was completely protected in all 288 sequences. In 99% of the DREB and ERF sequences, amino acids 27 W, 28 L, 29 G, 4 G, 8 R, 16E were completely protected. The sequences 37 A, 38 A were also highly protected.

Protein sequences of the two AP2 domains in AP2genes were found to have the following conserved amino acid residues in most of the sequences: 9 R, 12 G, 34Q, 38 G, 46 A, 47 A, in the first AP2 domain;1 R, 8 R, 9 G, 11 S, 15 G, and 26 W in the second domain. In this study, ERFs and DREBs were identified based on sequence alignment. The DREB and the ERF subfamilies were recognized from each other by the conserved amino acids 14 V and 19E in the former subfamily and 14Aand 19D in the latter. The researchers also classified the domains having observed 14 V as DREBs, irrespective of a residue in the 19th position due to the fact that 14 V has importance over 19E in determining the DNA-binding specificity of DREB transcription factor to the DRE cis-element^8^ (Fig. S1).

Interestingly, it was observed that in RAVs, ‘Glycine’ was found instead of ‘Valine’, and ‘Alanine’ was found conserved at position 14 in AP2/ERF proteins.

The sequences of the Soloist were HLG and LYD which have also been found in other plants like Arabidopsis.

### Gene structure of the AP2/ERF gene family

The members of *HaAP2/ERF* subfamily demonstrated similar exon-intron structures based on gene structure analysis. Generally, the number of exons ranged from 1 to 10, Soloist: 5 introns, RAV: 0-1 intron, AP2 and ANT: 4–9 introns, ERF and DREB: 0-1 intron. Of course, *HaAP2/ERF-182* had 2 introns, *HaAP2/ERF-215* and *HaAP2/ERF-217*had3 introns, and *HaAP2/ERF-214* contained 4 introns.

Some 86.29% of genes in the ERF subfamily were recognized as intronless, which was in agreement with a formerly published study^8^. On the other hand, the members of AP2 subfamily had more introns that the ERFs with at least five exons (Fig. S2). This extremely high variation in the gene structure suggests that a great differentiation may have occurred during the evolution of sunflower genome.

### Duplication and divergence rate of *HaAP2/ERF* genes

**Figure 5:**
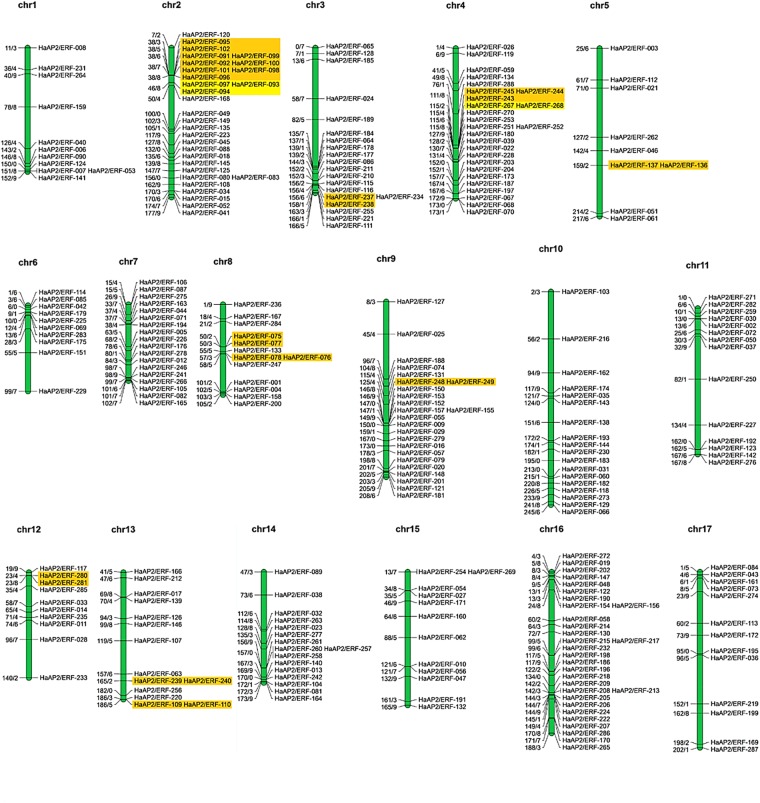
Distribution of 288 AP2/ERF genes on the 17 sunflower chromosomes. The tandemly duplicated gene pairs are indicated with yellow color.

**Figure 6:**
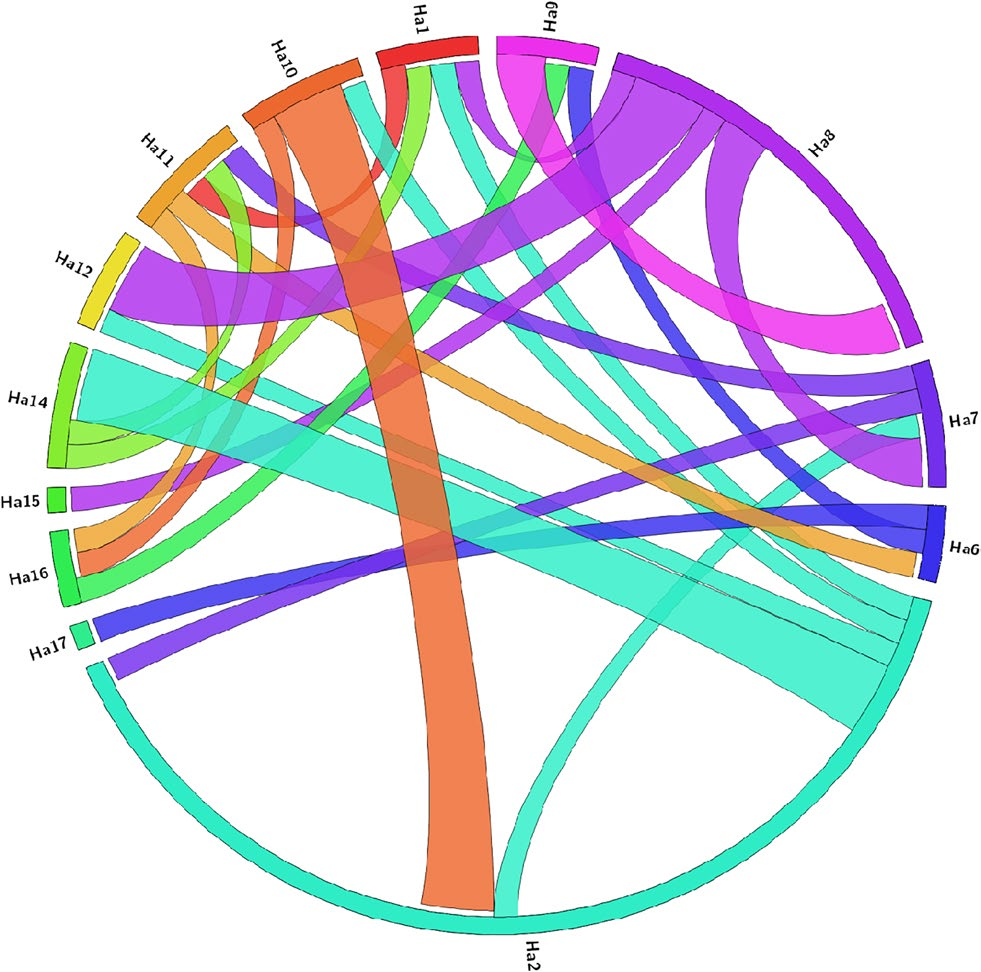
Distribution of segmentally duplicated *HaAP2/ERF* genes on *Helianthus annuus* chromosomes.

**Figure 7:**
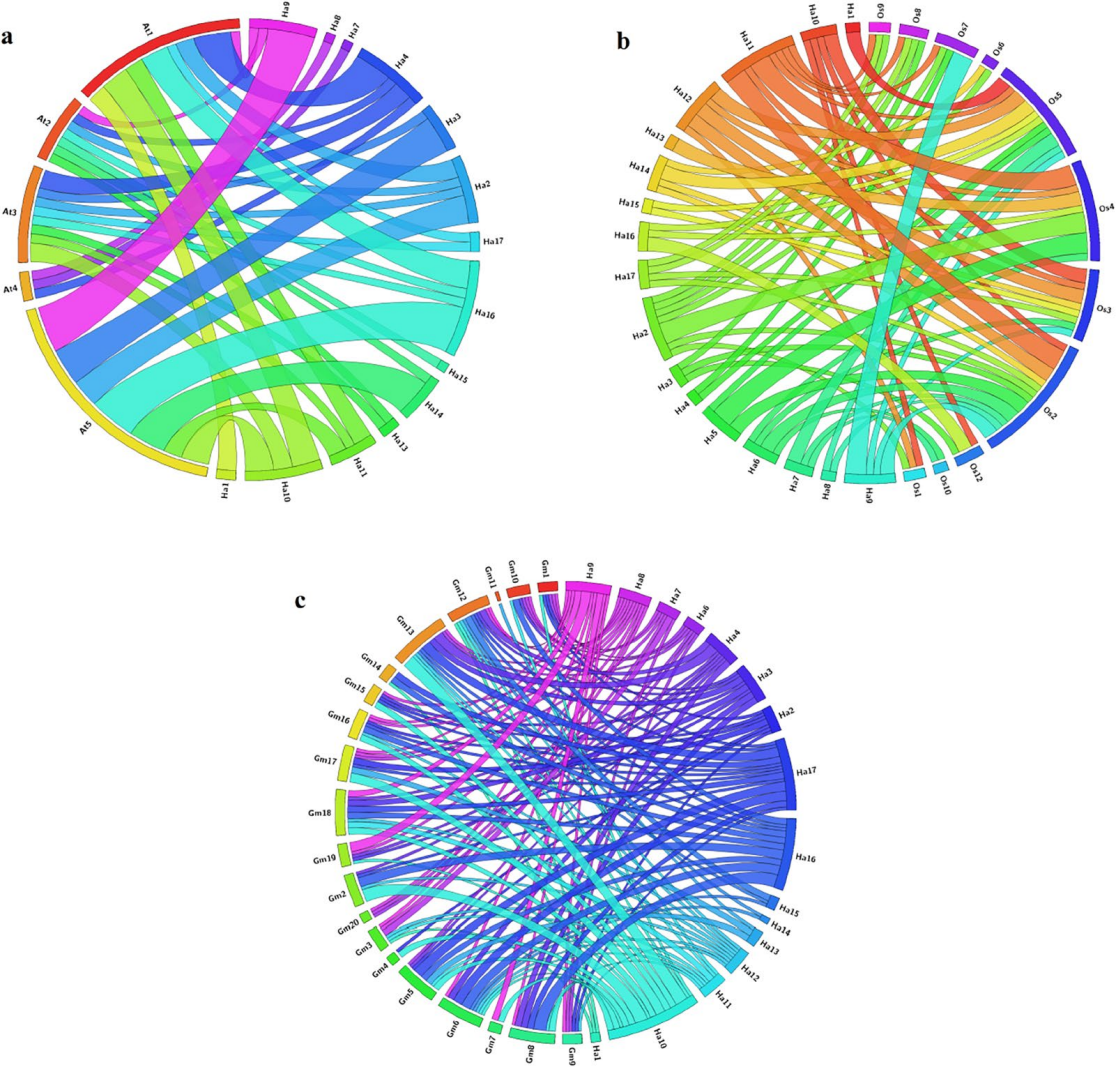
Comparative physical mapping showing the degree of orthologous relationships of *HaAP2/ERF* genes with (**a**) *Arabidopsis*, (**b**) rice, (**c**) soybean.

Analysis of synteny and gene duplication of AP2/ERFs among sunflower, Arabidopsis, soybean, and rice for the events of tandem and segmental duplication of *HaAP2/ERF* genes were surveyed through 17chromosomes of sunflower (Fig. 5). 288 AP2/ERF gene clusters contained 40 pairs of tandem duplicated genes located on chromosomes 2, 3, 4, 5, 8, 9, 12, and 13. Furthermore, 50 pairs of segment duplications were also identified(Fig. 6). In order to deduce the evolutionary origin of AP2/ERF genes, comparative syntenic analysis was performed among sunflower and Arabidopsis, soybean, and rice (Fig. 7a,b,c). Most of the *HaAP2/ERF* genes showed syntenic bias towards particular chromosomes of Arabidopsis, soybean and rice, which illustrated that the distribution and organization of AP2/ERF genes in these genomes have predominantly been shaped by the events of chromosomal reconstruction such as duplication and inversion.

Ka/Ks is an effective criterion for checking the positive selection pressure after duplication. Then if the Ka/Ks ratio = 1 means neutral selection, Ka/Ks < 1 represents pure selection and Ka/Ks > 1 represents the trend evolution accelerator with positive selection^9,55^. Additionally, the tandem and segmental duplications of the *HaAP2/ERF* genes were calculated to measure the influence of the selection (Tables S3 and S4). The Ka/Ks ratio for the pair of tandem duplication genes ranged from 0.05 to 1.33 with a mean of 0.42, while Ka/Ks for the segmental duplication was 0.03 to 1.81 with an average of 0.53. These results showed that duplicated genes of *HaAP2/ERF* were under the pressure of a strong purification selection by natural substitution and extensive selection constraints by natural selection during the evolution process. Additionally, such events of tandem and segmental duplication seem to have occurred around~2 to 101 Mya, respectively. Although tandem (Ka/Ks = 0.53) and segmental (Ka/Ks = 0.42) duplication of *HaAP2*/*ERF* genes are not similar under the positive evolutionary selection pressure, the duplication might have occurred simultaneously in both sets. In addition, the Ka/Ks ratio of orthologous gene pairs between sunflowers and the other three species was calculated (Tables S5, S6 and S7). The mean Ka/Ks was the highest between sunflowers and Arabidopsis (0.81), sunflowers and rice (0.81), and sunflowers and soybean (0.71), respectively, indicating that the genetic pairs between sunflowers and the studied species are strongly subjected to pure selection.

Divergence times were 52, 50 and 56 Mya for Arabidopsis, rice and soybeans, respectively. Therefore, we can conclude that tandem and segmental duplication events greatly contribute to the evolution and functional divergence of the AP2/ERF families of sunflowers and other species.

### Analysis of putative promoter regions of DREB gene subfamily

The regulatory elements of the *cis* play a key role in determining the characteristics of tissue or stress. In additions, the gene expression profiles have showed that multiple genes are closely correlated with *cis*-regulatory elements in their promoter sequences^56^. The upstream genes in 2000 bp greatly influence binding to target genes. In order to better understand the transcription rules and potential performance of the DREB subgenus genes in *Helianthus annuus*, 2000 bp justification zones were used to respond in terms of stress.

The regulatory *cis*-elements, i.e., multiple reproductive stress elements S000176, S000408 andS000415 for drought stress, S000453 for saline stress, S000030 for heat stress, S000407 for cold stress, and S000457 for wound stress were widely used in sunflower DREBs promoter regions as listed in Table S8. This clearly demonstrated that DREB is a transcription of the subcategory factors which can respond to abiotic stress and increase the potential functions in increasing acute abiotic resistance to stress. For example, *HaAP2/ERF-070* has a maximum of 28 drought stress elements (S000415) and *HaAP2/ERF-066* contains 34 elements of cold stress (S000407). Many studies on the performance of *HaDREB* genes provide a better understanding of the stress tolerance mechanism in sunflower.

### Gene ontology annotation

The GO analysis revealed, as presented in Table S9, the putative participation of *HaAP2/ERF* proteins in diverse biological, cellular and molecular processes. Annotation was performed on 288 *HaAP2/ERF* proteins and the results were described in 80 categories of biological processes. The analysis showed that predominant *HaAP2/ERF* proteins were involved in the regulation of transcription, i.e. the DNA-template process. This illustrated that the *HaAP2/ERF* proteins in association with the molecular functions were shown transcription factor activity and sequence-specific DNA binding. Prediction of cellular localization showed that the predominant 92% *HaAP2/ERF* proteins were localized in the nucleus. These are in concordance with formerly-reported experimental findings^10,45,57,58^.

### Gene expression and network interaction analysis

**Figure 8:**
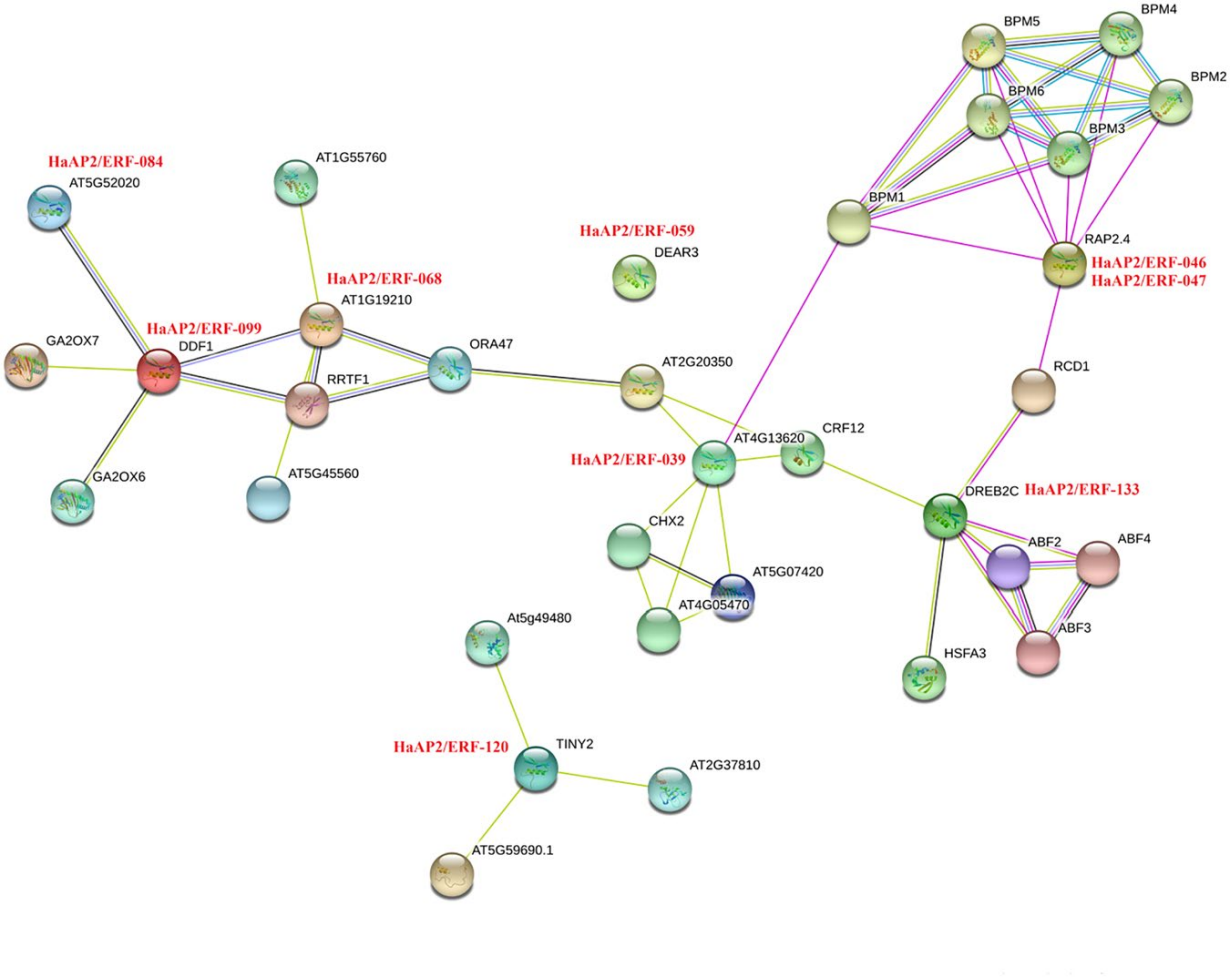
Interaction network of 9 *HaDREB* genes identified in sunflower and related genes in *Arabidopsis*.

Protein interactions in sunflower and Arabidopsis, including the functional physical interactions, were examined using STRING database for the identification of protein interactions. Nine proteins which displayed sequence similar to RAP2.4 (*HaAP2/ERF-04*6, *HaAP2/ERF-047*) and DEB2C (*HaAP/ERF-133*) were involved in a more powerful cross-linking network. *HaAP2/ERF-059*,which showed high coordination with DEAR3, was not involved in interactions. There were a large number of DREB and ERF types of stress sequence. We obtained 9 *HaDREB* family genes based on Arabidopsis protein interaction. To analyze the expression, real-time PCR was used to help us analyze the specifications of the family of CBF/DREB families under cold, salt, drought and heat stress conditions using two biological replicate. The accumulation in responses to abiotic stress for all genes analyzed (Fig. 8). In our study, we could find 9 novels *HaDREB* genes which showed different gene expression patterns under different treatment stress and control environment conditions (Table S10).

### Homology modeling of *HaAP2/ERF* proteins

**Figure 9:**
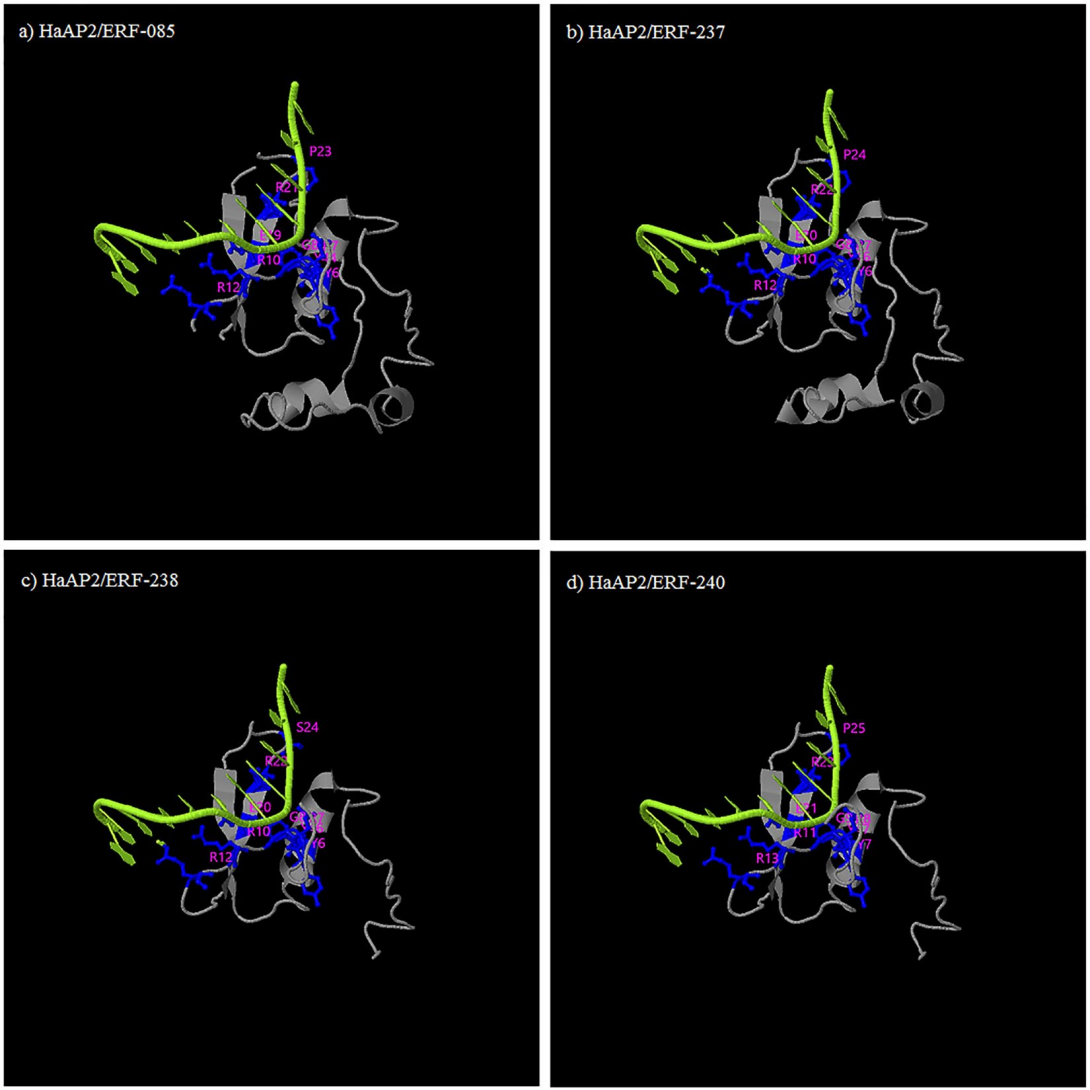
Predicated structures of *HaAP2/ERF* proteins. The structures of 4 *HaAP2/ERF* proteins with greater than 90% confidence level were shown along with its potential active site.

Using 3D protein models, four proteins were created by looking for a sequence similar to the PDB database using BLASTP. These four proteins were chosen because of their higher coordination with known protein sequences in PDB, and Phyre2 was used to model their predicted structure coordination. The protein structure of each of the four *HaAP2/ERFs* was modeled with 90% confidence and active potential sites were identified (Fig. 9). The 3D structure showed that the protection range of AP2/ERF contained about 50–60 amino acids in all *HaAP2/ERF* proteins with a typical three-dimensional compound to a layer of three antiparallel β-sheets followed by a parallel α-helix. Further examination of the AP2/ERF indicated the presence of YRG regions. The YRG region of 20 amino acids is a long-term N-terminal prolonged elongation at the base. Hydrophilic bases was reported to play an important role in direct communication with DNA^59^. AP2 subfamily members have two AP2/ERF domains separated by a linker sequence of 25 amino acids responsible for the placement of DNA binding domains. Molecular modeling has shown that all of the predicted protein structures are highly consistent and provide the basis for understanding the molecular sequence of *HaAP2/ERF* proteins.

### Expression profiles of *HaDREBs* under abiotic stress

**Figure 10:**
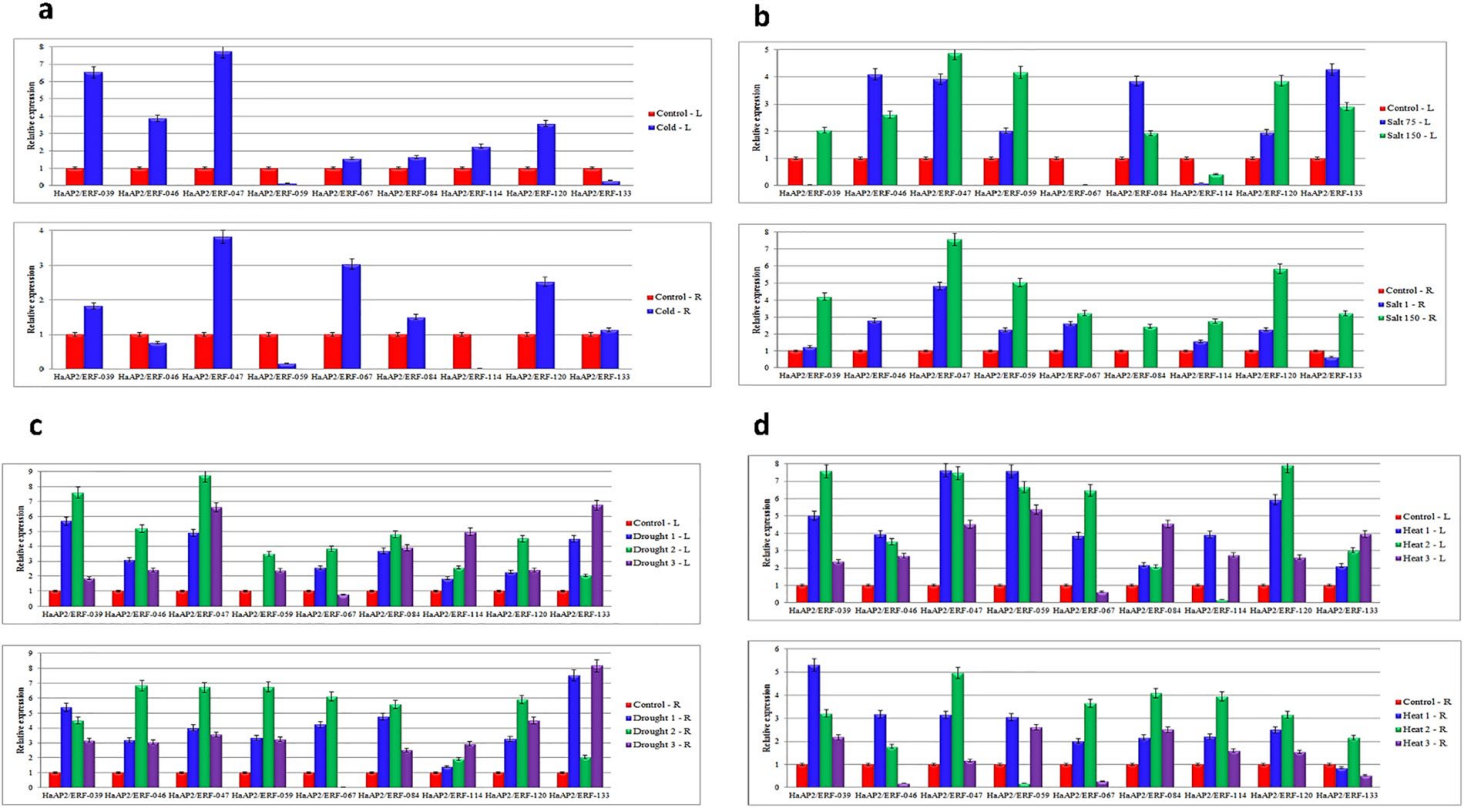
Relative quantitative (RQ) expression levels of 9 *HaDREB* genes at a series of time points following the abiotic stress treatments. (**a**) cold stress, (**b**) salt stress, (**c**) drought stress, (**d**) heat stress).

To further confirm the expression of these recognized AP2/ERF genes, 9*HaAP2/ERF* genes were randomly selected to help the researchers detect their expression levels in two tissues and under drought, cold, salt and heat treatments through qPCR (Fig. 10).

**Figure 11:**
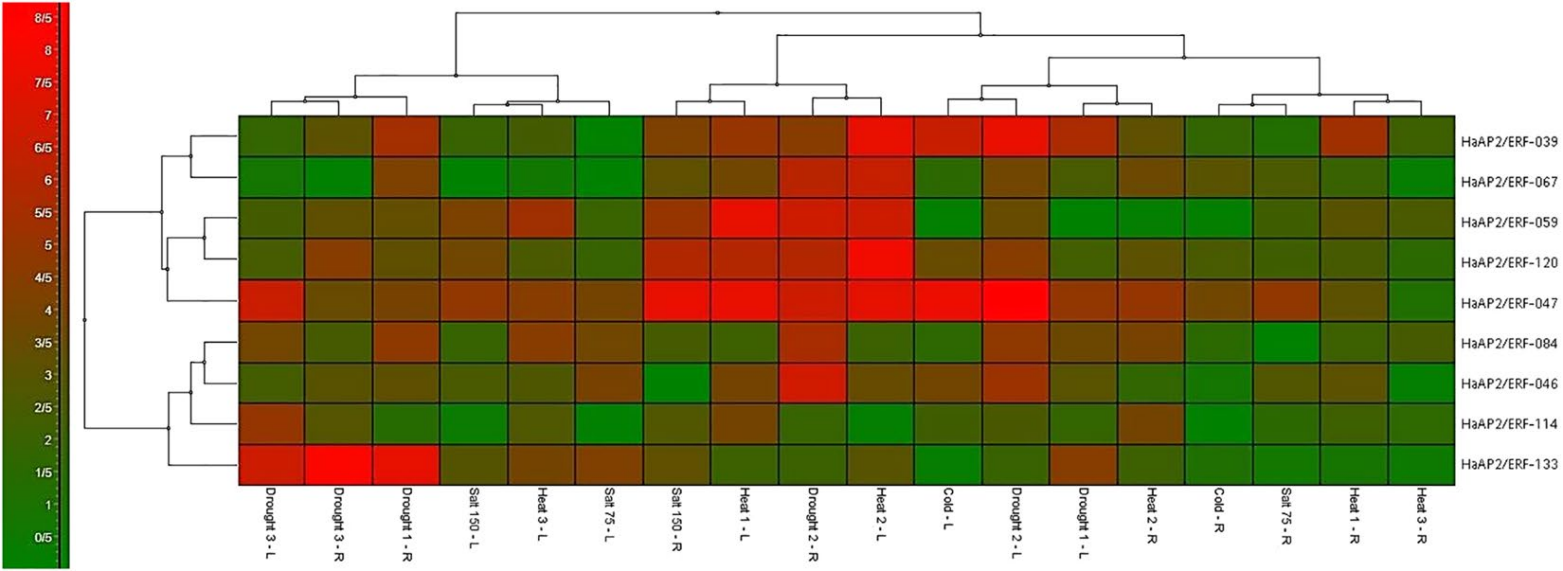
Heat map of the real-time quantitative PCR (qRT-PCR) analysis results of *HaDREB* genes in leaves and roots under drought, cold, heat and high salinity treatments, with three biological and technical replicates.

To understand the expressions of *HaDREB* transcription factors under abiotic stress, nine peptides were analyzed using the qRT-PCR method. To find out the expression of the genes, nine genes were randomly selected. Three genes belonged to group I, two genes belonged to group II, three genes belonged to group III and one gene belonged to group IV. During the experiments, it was found that the highest expression was associatedwith*HaAP2/ERF*-047gene and the lowest expression was found in gene *HaAP2/ERF*-114. *HaAP2/ERF*-047 and *HaAP2/ERF*-120 genes in all the applied abiotic stresses were up-regulating the effect of these two genes on sunflower resistance. Except for *HaAP2/ERF*-033, other genes were also up-regulated by increasing drought stress for 24 hours. However, after 48 hours from the onset of stress, they were down-regulated. Under cold stress, *HaAP2/ERF*-047 and *HaAP2/ERF*-039 were up-regulated, respectively, and played an effective role in resistance to cold stress. Yet,*HaAP2/ERF*-059 had a negative effect in cold stress. *HaAP2/ERF*-047 and *HaAP2/ERF*-120 can be considered as effective factors in salinity resistance due to their high expression in salinity stress. *HaAP2/ERF*-067 had a pronounced increase in heat stress conditions, but with passing of time, down-regulation of gene expression occurred in both leaf and root (Fig. 11).

### Determination of proline content

In this experiment, proline was studied under drought, salinity, cold and heat stress. The results indicated that proline was increased under abiotic stress. The highest amount of proline in leaf and root is in drought stress after 48 hours. After 6 hours of heat treatment, the lowest change occurred in control conditions. In the cold stress, the proline increased. Also, in salinity and heat stresses, the amount of proline increased in leaves and roots by increasing the levels of stress, which indicates the response of the plant to the stress and that this response helps preserve the plant under stress conditions (Fig. S3).

### Relative water content (RWC) and Na^+^/K^+^

The effects of various saltiness stresses on physiological and genetic attributes of plants were assessed by measuring the progressions in RWC and sodium and potassium fixation in leaf and root under stress. The variation of Na^+^/K^+^ was astoundingly higher in root tissues as compared with leaf (Fig. S4). The average of RWC of plants in response to drought stress demonstrated a slight decrease at the earlier stage of stress, beginning to lessen strongly afterwards (Fig. S5). The outcomes demonstrated that plants were, altogether influenced by abiotic stress.

## Discussion

The AP2/ERF is one of the major families of transcriptional factor plants. They plays an important role in transcription regulation, which involves complex growth processes, biological stresses, seed germination, flower growth, aging, fruit arrival, response to salt, drought, low temperature, and pathogen attack^39,60–64^. Compared to other species, AP2/ERF in sunflower is much larger than rice (174genes) and Arabidopsis (148genes)^10^. A protected motif is a sequence of amino acids protected by a variety of biological functions and can be involved in transcriptional activities, proteins, and nuclear positions^10^. Similar motifs and functions are observed in the proteins which are categorized in a subgroup. Researchers have identified various preservative patterns in Arabidopsis family AP2/ERF and rice^10^. In this study, we have a form of each group within the family of *PeAP2/ERF* gene compared to the family of *AtAP2/ERF* family. It is known that the family of AP2/ERF gene is significantly based on the number of ERF members^65^. It has been found that 248 members are in the family of ERF in sunflower. However, this number is 122 and 139 in Arabidopsis and rice, respectively. Also, 35 genes were attributed to the AP2 subfamily, which represents 18 and 26 plants in the Arabidopsis and rice plants, respectively^10^. In contrast, the number of RAV family members did not significant among 4, 6 and 7 of sunflower, Arabidopsis and rice^10^, respectively. Therefore, the frequency of the upperAP2/ERF gene in sunflower may be due to the high number of members in ERF and DREB subfamily. It has been widely recognized that AP2/ERF transcription factors play an important role in regulating the growth, evolution and response of plants to various stresses as a signal transmission pathway in plants^66^. However, the *HaAP2/ERFs* performance is not well understood at the moment. In the current experimental research, gene expressions of patterns in different plant tissues under various stress conditions were studied to help clearly understand their potential capability during environmental tensions. We recognized that ERF gene family had fewer introns than the AP2 family in sunflower, which may have faster response and expression of most ERF genes during development^65^. AP2/ERF proteins can link to the GCC-box or DRE through the ERF domain and then express the target gene under stress conditions^67,68^. Among them, *HaAP2/ERF-047*, a member of the subfamily of the DREB family, is significantly controlled by both cold and drought stress. In addition, a total of 8 proteins, S000415 (elemental response to dehydration), 14S000407 (cold responsive element), and 11 S000453 (salt-responding element) of *cis*-elements in the *HaAP2/ERF-047* promoter region were identified. Moreover, *HaAP2/ERF-047*, *HaAP2/ERF-039* and *HaAP2/ERF-120* were significantly up-regulated under studied abiotic stress. We predicted that *cis-*elements were vital regimens for controlling the expression of *HaAP2/ERF* which respond to other functional proteins with an AP2/ERF transcription factor in order to create a complex regulatory metabolic network throughout developmental processes and stress conditions. Previous studies showed that DREB and ERF included protected WLG motifs in AP2/ERF^10,69^. In this research, the WLG motif in the DREB and ERF subfamilies were very well protected (Fig. S1). While the 14^th^alanine and the 19^th^aspartic acid are conserved in the ERF proteins, valine and glutamic acid are conserved within DREB proteins^10^. Two protected amino acids on the putty are β-sheet in AP2/ERF gene family, which like to bind to DNA sequences^8^.

Significantly, all subgroups of DREB and ERF were completely protected in the amino acid residues of Val-14 and Ala-14, respectively. These preserved amino acid residues are likely to play an important role for the genes of the DREB/ERF family subfamily that are involved in various forms of physical interaction with DNA^8^.Recurring events on a large scale are defined as the simultaneous repetition of genes. Ka and Ks are steps to discover the mechanism of gene deviation after replication. It is expected that in a molecular clock, Ks of repetition is expected to be similar over time. However, there are significant changes among genes^70^. For a better description of evolutionary patterns, estimates of evolutionary rates are very useful^71^.

Time (million years ago, Mya) of duplication and divergence was calculated using non-synonymous mutation rate of one substitution per synonymous site per year as T = Ks/2λ (λ = 6.5 × 10–9)^55,72^. We estimated the divergence between sunflower and Arabidopsis, and rice and soybeans, the value of Ka/Ks (the ratio of the amount of substitution unknown to the amount of synonymous replacement), and the ratio of the height of the species compared to the figures above, which indicated a strong selection pressure on these genes. If the amino acid replacement with the same synonymous equations occurs, after several amino acid replacement reactions, the gene was removed from the copy, meaning that Ka/Ks = 1. In other words, duplicate genes had selective or limited constraints. If Ka/Ks < 1, after replacing the replacement by natural selection, perhaps because of the deleterious effects, the smaller Ka/Ks represents a greater selective limitation and the number of removed substitutions in which the two genes evolved^73,74^. The mean Ka/Ks indicated a pair of genes between sunflower and Arabidopsis (0.81), sunflower and rice (0.81), and sunflower and soybean (0.71). This indicated that a specific texture and strategy derived from *HaAP2/ERF* provide valuable candidates for more applied studies on AP2/ERF genes in *Helianthus annuus* as well as in other oil-seed plants.

## Conclusions

The current study aimed at identifying and characterizing the AP2/ERF transcription factors in *Helianthus annuus*. By conducting an extensive genome search, 288 *HaAP2/ERF* genes were obtained. Visiting EST or complete cDNA sequences confirmed all of their facts. The location of the chromosomes, the exon-intron structure, the protective motif combination, and the phonological relationship of *HaAP2/ERFs* were analyzed and compared. *HaAP2/ERFs* can be categorized into four sub-groups with regard to the number of AP2 domains and probabilistic functions. Gene expression of *HaAP2/ERF* genes in different tissues (leaf and root) and also when exposed to heat, cold, salt and drought stress were studied. Several *HaAP2/ERF* genes were identified that could be considered as a candidate for a further study on their performance in plant growth and stress response. This study, for the first time, provides the organization, structure, evolution and expression of the *HaAP2/ERF* family, which facilitates the analysis of the *HaAP2/ERF* gene function analysis and establishes a basis for a better understanding of the molecular mechanism of plant development and physiological stress processes in *Helianthus annuus*.

## Methods

### Sequence recovery and recognition of AP2/ERF gene family in sunflower genome

The entire genome information of *Helianthus annuus* was available at sunflower database (https://www.heliagene.org). The researchers downloaded the anticipated protein sequences as the dataset for downstream analysis (v1.0.29). The AP2/ERF domain (PF00847) obtained from PFAM database (http://pfam.xfam.org/) was utilized as the question for Hidden Markov Model(HMM) look utilizing HMMER 3.0 program with a pre-characterized limit of E < 1e^−5^. Moreover, the plant transcription factor database (http://plntfdb.bio.uni-potsdam.de/v3.0/) was used to take the AP2/ERF protein arrangements of Arabidopsis and then utilized as query to search against the *Helianthus annuus* protein dataset using the BLASTP program with an estimation of 1e^−5^ and 50% as the threshold. Besides, HMMER and BLAST hits were analyzed and parsed. Afterwards, a self-blast of these sequences was performed in order to remove the redundancy without considering any alternative splice variants. After manual amending, the putative *HaAP2/ERF* proteins were obtained. At that point, the NCBICDD web server (http://www.ncbi.nlm.nih.gov/Structure/cdd/wrpsb.cgi) and SMART database (http://smart.embl-heidelberg.de/site) were utilized to further confirm the anticipated *HaAP2/ERF* genes.

Then, the analysis of the composition as well as the physical/chemical characterization of the sunflower AP2/ERFs (number of amino acids, molecular weight, and pI) was conducted. Protein statistics were analyzed using the Sequence Manipulation Suite (http://www.bio-soft.net/sms/). Finally, Softberry (http://linux1.softberry.com/) was used for the prediction of subcellular localization.

### Phylogenetic analysis

Clustal X ver.2.1 was utilized to perform multiple sequence alignment^75^. Also, an un-rooted neighbor joining (NJ) tree with 1000 bootstrap replications was constructed using MEGA ver.7.0^76^.

### Chromosome distribution, gene structure and conserved motif analysis

The chromosome distributions of these genes were obtained from the genome annotation information and then validated by BLASTN search. The Gene Structure Display Server (http://gsds.cbi.pku.edu.cn/) was deployed to show the exon-intron organizations of the predicted AP2/ERF genes. Conserved motifs or domains were predicted using the MEME Suite web server (http://meme-suite.org/). The physical distribution of AP2/ERF genes on chromosomes was drawn by MapChart based on gene position in the genome^77^.

### Gene duplication, orthologous analysis, evolutionary patterns, and divergence of AP2/ERF gene family

To identify the contribution of segmental and tandem gene duplications in genome-wide expansion of AP2/ERF family in the considered *Helianthus annuus*, genes which were found within 5-Mb regions with 80% and higher similarity with e-value threshold of 1e^−10^ were considered as tandemly duplicated genes, and the ones separated by >5 Mb distance were identified as segmentally duplicated genes^78^.

The occurrence of duplication events, the divergence of homologous genes, and the selective pressure on duplicated genes were estimated by calculating synonymous (Ks) and non-synonymous substitutions (Ka) per site between the duplicated gene-pairs using DnaSPver.5.10.1^79,80^. Time (million years ago, Mya) of duplication and divergence were calculated using asynonymous mutation rate of λ substitutions per synonymous site per year as T = Ks/2λ (λ = 6.5 × 10^−9^)^55,71^.

For synteny analysis, duplications between *Helianthus annuus*AP2/ERF genes as well as the synteny block of this family among sunflower and other three species (Arabidopsis, rice and soybean) were obtained from PGDBj and the diagrams were drawn by Circos ver.0.67^81^.

### Analysis of putative promoter regions of DREB gene subfamily in *Helianthus annuus*

The upstream 2000-bp genomic DNA sequences of all recognized AP2/ERF genes were downloaded from the *Helianthus annuus* genome. They were then submitted to PLACE database (http://www.dna.affrc.go.jp/PLACE/) for the purpose of identifying *cis*-regulatory elements in the promoter regions.

### AP2/ERF protein annotations and interaction networks

Gene ontology (GO) analysis was applied to predict gene functions and to calculate the frequency of functional categories based on the sequences obtained. Blast2GO software (https://www.blast2go.com/)^82^ was used to determine the GO annotations. The GO terms for each of the three main categories (biological process, molecular function, and cellular component) were obtained from sequence similarity using the default parameters. Network interaction analysis data related to the nine genes in DREB of sunflower were obtained from STRING online database (https://string-db.org/)^83^.

### Homology modeling of *HaAP2/ERF* proteins

For homology modeling, all the *HaAP2/ERF* proteins were queried against the Protein Data Bank (PDB)^84^ to identify the best template with a similar amino acid sequence and a known 3-D structure. The data was fed in Phyre2 server (Protein Homology/AnalogY Recognition Engine; http://www.sbg.bio.ic.ac.uk/ phyre2) for the prediction of the 3-D structure of proteins by homology modeling under ‘normal’ mode^85^. COACH server (http://zhanglab.ccmb.med. umich.edu/COACH/) and UCSF Chimera 1.8 were used to predict the active site and to highlight it, respectively.

### Plant materials, growth conditions and stress treatments

Seeds of sunflower cultivar ‘Fantasia’ were obtained from Agriculture Research Institute (ARI), Safie-Abad Dezful, Iran. The seeds were sown in composite soil (peat compost: vermiculite: sand, 2:2:1) in the glasshouse at Shahid Chamran University of Ahvaz, Iran at 28 ± 1 °C day/23 ± 1 °C night temperature with 70 ± 5% relative humidity and natural sunlight during June–July, 2017. Roots and leaves were collected from ‘Fantasia’ genotype for RNA isolation and organ-specific analysis.

### Drought stress

Four -week-old plantlets (6 leaf stages) of sunflower were subjected to water stress by withholding water for 12, 24 and 48 h^86^. To determine the plant water status, the relative water content (RWC) was measured in leaves of the samples via Catsky method^87^.

### Salt stress

Plantlets were moved into solutions containing either 75 or150 mM of NaCl^87,88^. Leaf and root samples from the stressed plants were collected 24 h later alongside the control samples. Potassium to sodium ratio was measured as the criterion for examining different salinity stress levels^87^.

### Heat stress

Four-week-old plantlets in strength Hoagland’s solution were transferred to humidity growth chamber (Memmert, Germany) with 70% relative humidity and maintained at 42 ± 1 °C for1.5, 3 and 6 h^78,87^.

### Cold stress

For low-temperature stress treatments, plants were transferred to an illuminated incubator at 4 °C with other culture conditions unchanged. After two hours, the leaves and roots were collected for analysis. The roots were rapidly washed with distilled water (4 °C incubated water for low- temperature treatment)^89^.

The plants were supplemented with water and Hoagland’s solution on alternate days. Unstressed plants were maintained as the control group. After stress treatments, whole seedlings were carefully harvested, immediately frozen in liquid nitrogen and stored at −80 °C until RNA isolation. For precision and reproducibility concerns, the researchers conducted three independent experiments in each of which 100 mg seedling samples were collected by random sampling.

### Determination of proline content

The same fresh samples which were utilized for gene expression analysis were used to determine the proline content in the samples. Each treatment had three pot replications and the sample from each pot was mixed together as replications. Free proline content was determined by ninhydrin assay at A520 nm in line with the method described by Bates *et al*.^90^.

### RNA extraction and expression analysis using qRT-PCR

Total RNA was isolated from leave and root samples (abiotic stress and control sunflower cv. ‘Fantasia’ seedlings) using BioBasic RNA extraction kit (BS82314-BioBasic, Canada) following the manufacturer’s instructions.DNA contamination was removed from the RNA samples using RNase-free DNaseI (1 U ml2l, TaKaRa, Dalian, China). The quality and purity of the RNA preparations were determined by measuring the OD260/OD280 absorption ratio (1.9–2.0), and the integrity of the preparations was determined by electrophoresis in a 1.2% agarose gel containing formaldehyde as described in previous studies^91,92^. RNA concentrations were measured by a spectrophotometer (Eppendorf, USA). About 1 mg of total RNA was used to synthesize first strand cDNA primer with OligodT in a 20 ml reaction mix using 200 U/ml of PrimeScript M-MuLV reverse transcriptase (Takara Bio Inc., USA) following the manufacturer’s instructions. Quantitative real time (qRT) PCR was performed using SYBR Premix ExTaq II (TliRNaseH Plus) (Takara Bio Inc., USA) on Master cycler system (ABI, Biosystem, USA) in triplicate^93^. The constitutive gene *Actin* (Gene Bank ID: AF282624.1.) and *GAPDH* (Gene Bank ID: DQ503718.1) from sunflower were used as endogenous control.

qPCR was used to resolve the transcript levels of nine randomly selected *HaAP2/ERF* genes according to EbrahimiKhaksefidi *et al*.^86^ and Chen *et al*.^94^. The primers are listed in Table S11.

### Data availibity

Availability of data and all material of the datasets supporting the results of this article are included within the article and its supplementary files.

## Electronic supplementary material

Supplementary Figures

Supporting Information Files

Supplementary Table S

## References

[1] Kamiloglu O, Ercisli S, Sengul M, Toplu C, Serce S (2009). Total phenolics and antioxidant activity of jujube (*Zizyphus jujube* Mill.) genotypes selected from Turkey. Afri. J Biotechnol..

[2] Tosun M, Ercisli S, Karlidag H, Sengul M (2009). Characterization of red raspberry *(Rubusidaeu*s L.) genotypes for their physicochemical properties. J. Food Sci..

[3] Tahtamouni R, Shibli R, Al-Abdallat A, Al-Qudah T (2016). Analysis of growth, oil yield, and carvacrol in *Thymbra spicat*a L. after slow-growth conservation. Turk. J. Agric. For..

[4] Cuce M, Sokmen A (2017). *In vitro* production protocol of *Vaccinium uliginosum* L. (bog bilberry) growing in the Turkish flora. Turk. J. Agric. For..

[5] Thamilarasan SK, Park JI, Jung HJ, Nou IS (2014). Genome-wide analysis of the distribution of AP2/ERF transcription factors reveals duplication and CBFs genes elucidate their potential function in *Brassica olerace*a. BMC Genomics..

[6] Guo B (2016). Genome-Wide Analysis of APETALA2/Ethylene-Responsive Factor (AP2/ERF) Gene Family in Barley (*Hordeum vulgare* L.). PLoS ONE..

[7] Lata C (2014). Genome-Wide Investigation and Expression Profiling of AP2/ERF Transcription Factor Superfamily in Foxtail Millet (*Setaria italica* L.). PLoS ONE..

[8] Sakuma Y (2002). DNA-binding specificity of the ERF/AP2 domain of Arabidopsis DREBs, transcription factors involved in dehydration and cold-inducible gene expression. Biochem Biophys Rese Commun..

[9] Cui L (2016). Genome-wide identification, phylogeny and expression analysis of AP2/ERF transcription factors family in *Brachypodium distachyon*. BMC Genomics..

[10] Nakano T, Suzuki K, Fujimura T, Shinshi H (2006). Genome-wide analysis of the ERF gene family in Arabidopsis and rice. Plant Physiol..

[11] Jofuku KD, Den Boer B, Van Montagu M, Okamuro JK (1994). Control of Arabidopsis flower and seed development by the homeotic gene APETALA2. Plant Cell..

[12] Elliott RC, Betzner AS, Huttner E (1996). AINTEGUMENTA, an APETALA2-like gene of Arabidopsis with pleiotropic roles in ovule development and floral organ growth. Plant Cell.

[13] Song X, Li Y, Xilin Hou X (2013). Genome-wide analysis of the AP2/ERF transcription factor superfamily in Chinese cabbage (*Brassica rapa ssp. pekinensis*). BMC Genomics..

[14] Hao D, Ohme-Takagi M, Sarai A (1998). Unique mode of GCC box recognition by the DNA-binding domain of ethylene-responsive element-binding factor (ERF domain) in plant. J.Biol Chem..

[15] Kagaya Y, Ohmiya K, Hattori T (1999). RAV1, a novel DNA-binding protein, binds to bipartite recognition sequence through two distinct DNA-binding domains uniquely found in higher plants. Nucleic Acids Res..

[16] Krizek B (2009). AINTEGUMENTA and AINTEGUMENTA-LIKE6 act redundantly to regulate Arabidopsis floral growth and patterning. Plant Physiol..

[17] Xu ZS, Chen M, Li LC, Ma YZ (2011). Functions and application of the AP2/ERF transcription factor family in crop improvement. J. Integr. Plant Biol..

[18] Gutterson N, Reuber TL (2004). Regulation of disease resistance pathways by AP2/ERF transcription factors. Curr. Opin. Plant Biol..

[19] Aukerman MJ, Sakai H (2003). Regulation of flowering time and floral organ identity by a microRNA and its APETALA2-like target genes. Plant Cell.

[20] Taketa S (2008). Barley grain with adhering hulls is controlled by an ERF family transcription factor gene regulating a lipid biosynthesis pathway. Proc. Natl. Acad. Sci. USA.

[21] Jofuku KD, Omidyar PK, Gee Z, Okamuro JK (2005). Control of seed mass and seed yield by the floral homeotic gene APETALA2. Proc. Natl. Acad. Sci. USA.

[22] Alonso JM (2003). Genome-wide insertional mutagenesis of *Arabidopsis thaliana*. Science.

[23] Hu YX, Wang YH, Liu XF, Li JY (2004). Arabidopsis RAV1 is down-regulated by brassinosteroid and may act as a negative regulator during plant development. Cell Res..

[24] Sohn KH, Lee SC, Jung HW, Hong JK, Hwang BK (2006). Expression and functional roles of the pepper pathogen-induced transcription factor RAV1 in bacterial disease resistance, and drought and salt stress tolerance. Plant Mol. Biol..

[25] Li CW (2011). Tomato RAV transcription factor is a pivotal modulator involved in the AP2/EREBP mediated defense pathway. Plant Physiol..

[26] Ito Y (2006). Functional analysis of rice DREB1/CBF-type transcription factors involved in cold-responsive gene expression in transgenic rice. Plant Cell Physiol..

[27] Jaglo-Ottosen KR, Gilmour SJ, Zarka DG, Schabenberger O, Thomashow MF (1998). Arabidopsis CBF1 over expression induces COR genes and enhances freezing tolerance. Science.

[28] Qin F (2007). Regulation and functional analysis of ZmDREB2A in response to drought and heat stresses in *Zea mays* L. Plant J..

[29] Hong JP, Kim WT (2005). Isolation and functional characterization of the CaDREBLP1 gene encoding a dehydration responsive element binding-factor-like protein 1 in hot pepper (*Capsicum annuum* L. cv. Pukang). Planta.

[30] Vendruscolo ACG (2007). Stress-induced synthesis of proline confers tolerance to water deficit in transgenic wheat. J. Plant Physiol..

[31] Bohenert HJ, Shen B (1999). Transformation and compatible solutes. Sci.Horti..

[32] Lazcano-ferrat I, Lovatt CJ (1999). Relationship between relative water content, nitrogen pools and growth of *Phaseolus vulgaris* L. and *P. acutifolius* A. Gray during water deficit. Crop Sci..

[33] Merah O (2012). Genetic analysis of phytosterol content in sunflower seeds. Theor. Appl. Genet..

[34] Hong B (2009). Over-expression of AtDREB1A in *Chrysanthemum enhances* tolerance to heat stress. Plant Mol. Biol..

[35] Wang XM (2011). CkDREB gene in *Caragana korshinskii* is involved in the regulation of stress response to multiple abiotic stresses as an AP2/EREBP transcription factor. Mol. Biol. Rep..

[36] Liang CL (2012). Immunotoxicologic assessment of genetically modified drought-resistant wheat T349 with GmDREB1. Zhonghua Yu Fang Yi XueZaZhi..

[37] Cheng MC, Liao PM, Kuo WW, Lin TP (2013). The Arabidopsis ethylene response factor1 regulates abiotic stress-responsive gene expression by binding to different *cis*-acting elements in response to different stress signals. Plant Physiol..

[38] Fujita Y, Fujita M, Shinozaki K, Yamaguchi-Shinozaki K (2011). ABA-mediated transcriptional regulation in response to osmotic stress in plants. J. Plant Res..

[39] Wang Q (2008). Overexpression of a rice OsDREB1F gene increases salt, drought, and low temperature tolerance in both Arabidopsis and rice. Plant Mol. Biol..

[40] Mallikarjuna G, Mallikarjuna K, Reddy MK, Kaul T (2011). Expression of OsDREB2A transcription factor confers enhanced dehydration and salt stress tolerance in rice (*Oryza sativa* L.). Biotech. Letters.

[41] Dubouzet JG (2003). OsDREB genes in rice, *Oryza sativa* L., encode transcription activators that function in drought-, high-salt- and cold-responsive gene expression. Plant J. Cell & Mol.Biol..

[42] Novillo F, Alonso JM, Ecker JR, Salinas J (2004). CBF2/DREB1C is a negative regulator of CBF1/DREB1B and CBF3/DREB1A expression and plays a central role in stress tolerance in Arabidopsis. Proc. Natl. Acad.Sci. USA.

[43] Nakashima K (2000). Organization and expression of two Arabidopsis DREB2 genes encoding DRE-binding proteins involved in dehydration- and high-salinity-responsive gene expression. Plant Mol.Biol..

[44] Liu L (2008). Molecular cloning, expression profiling and trans-activation property studies of a DREB2-like gene from chrysanthemum (*Dendranthe mavestitum*). J. Plant Rese..

[45] Chen J, Xia X, Yin W (2009). Expression profiling and functional characterization of a DREB2-type gene from *Populus euphratica*. Bioch. Bioph. Rese. Comm..

[46] Gupta K, Agarwal PK, Reddy MK, Jha B (2010). SbDREB2A, an A-2 type DREB transcription factor from extreme halophyte *Salicornia brachiata* confers abiotic stress tolerance in *Escherichia coli*. Plant Cell Rep..

[47] Wu H (2015). Genome-wide analysis of the AP2/ERF transcription factors family and the expression patterns of DREB genes in Moso Bamboo (*Phyllosta chysedulis*). PLoS One.

[48] Licausi F (2010). Genomic and transcriptomic analysis of the AP2/ERF superfamily in *Vitis vinifera*. BMC Genomics.

[49] Du H, Huang M, Zhang Z, Cheng S (2014). Genome-wide analysis of the AP2/ERF gene family in maize water logging stress response. Euphytica.

[50] Zhang C (2012). Genome-wide analysis of the AP2/ERF superfamily in peach (*Prunus persica*). Genet Mol Res..

[51] Rashid M, Guangyuan H, Guangxiao Y, Hussain J, Xu Y (2012). AP2/ERF transcription factor in rice: genome-wide canvas and syntenic relationships between monocots and eudicots. Evol. Bioinfo. Online.

[52] Vogel JP (2010). Genome sequencing and analysis of the model grass *Brachypodium distachyon*. Nature.

[53] Wu ZJ (2015). Transcriptome-based discovery of AP2/ERF transcription factors related to temperature stress in tea plant (*Camellia sinensis*). Funct. Integr. Genomics.

[54] Liu L, White MJ, MacRae TH (1999). Transcription factors and their genes in higher plants. Eur. J. Biochem..

[55] Lynch M, Conery JS (2000). The evolutionary fate and consequences of duplicate genes. Science.

[56] Fang YJ, You J, Xie KB, Xie WB, Xiong LZ (2008). Systematic sequence analysis and identification of tissue-specific or stress-responsive genes of NAC transcription factor family in rice. Mol. Genet. Genomics.

[57] Lata, C., Yadav, A. & Prasad, M. *Role of plant transcription factors in abiotic stress tolerance*. In: Shanker A. and Venkateshwarulu B. (eds) Abiotic Stress Response in Plants, INTECH Open Access Publishers, 269–296 (2011).

[58] Sun ZM, Zhou ML, Xiao XG, Tang YX, Wu YM (2014). Genome-wide analysis of AP2/ERF family genes from *Lotus corniculatus* shows LcERF054 enhances salt tolerance. Funct. Integr. Genomics.

[59] Okamuro JK, Caster B, Villarroel R, van Montagu M, Jofuku KD (1997). The AP2 domain of APETALA2 defines a large new family of DNA binding proteins in *Arabidopsis*. Proc. Natl. Acad. Sci. USA.

[60] Wolfe SA, Nekludova L, Pabo CO (2000). DNA recognition by Cys2His2 zinc finger proteins. Annu. Rev. Biophys. Biomol. Struct..

[61] Schmidt R (2013). SALT-RESPONSIVEERF1 regulates reactive oxygen species-dependent signaling during the initial response to salt stress in rice. Plant Cell.

[62] Zhu XL (2014). The wheat ethylene response factor transcription factor pathogen-induced ERF1 mediates host responses to both the necrotrophic pathogen *Rhizoctonia cerealis* and freezing stresses. Plant Physio..

[63] Klucher KM, Chow H, Reiser L, Fischer RL (1996). The AINTEGUMENTA gene of Arabidopsis required for ovule and female gametophyte development is related to the floral homeotic gene APETALA2. Plant Cell.

[64] Yang CY, Hsu FC, Li JP, Wang NN, Shih MC (2011). The AP2/ERF transcription factor AtERF73/HRE1 modulates ethylene responses during hypoxia in *Arabidopsi*s. Plant Physio..

[65] Shu Y, Liu Y, Zhang J, Song L, Guo C (2016). Genome-wide analysis of the AP2/ERF superfamily genes and their responses to abiotic stress in *Medicago truncatula*. Front. Plant Sci..

[66] Mizoi J, Shinozaki K, Yamaguchi-Shinozaki K (2012). AP2/ERF family transcription factors in plant abiotic stress responses. Biochim. Biophys. Acta..

[67] Shigyo M, Hasebe M, Ito M (2006). Molecular evolution of the AP subfamily. Gene.

[68] Lata C, Prasad M (2011). Role of DREBs in regulation of abiotic stress responses in plants. J. Exp. Bot..

[69] Liu S (2013). Genome-Wide analysis of *ZmDREB* genes andtheir association with natural variation in drought tolerance at seedling stage of *Zea mays* L. Plos Genetics.

[70] Shiu SH (2004). Comparative analysis of the receptor-like kinase family in Arabidopsis and rice. Plant Cell.

[71] Cao J, Huang JL, Yang YP, Hu XY (2011). Analyses of the oligo peptide transporter gene family in poplar and grape. BMC Genomics.

[72] Yang Z (2008). Molecular evolution of the cpp-like gene family in plants: insights from comparative genomics of Arabidopsis and rice. J. Mol. Evol..

[73] Lynch OT, Giembycz MA, Daniels I, Barnes PJ, Lindsay MA (2000). Pleiotropic role of lyn kinase in leukotriene B4-induced eosinophil activation. Blood.

[74] Wagner A (2002). Selection and gene duplication: a view from the genome. Genome Biol..

[75] Larkin MA (2007). Clustal W and clustal X version 2.0. Bioinformatics.

[76] Tamura K, Stecher G, Peterson D, Filipski A, Kumar S (2013). MEGA6: molecular evolutionary genetics analysis version 6.0. Mol. Biol. Evol..

[77] Voorrips RE (2002). MapChart: Software for the graphical presentation of linkage maps and QTLs. J.Hered..

[78] Agarwal, G. *et al*. Genome-wide dissection of AP2/ERF and HSP90 gene families in five legumes and expression profiles in chickpea and *pigeon pea*. *Plant Biotech. J*. 1–15 (2016) 10.1111/pbi.12520 PMC5066796 26800652

[79] Librado P, Rozas J (2009). DnaSP v5: A software for comprehensive analysis of DNA polymorphism data. Bioinformatics.

[80] Rozas, J. *DNA Sequence Polymorphism Analysis using DnaSP*. In Posada D. (ed.) Bioinformatics for DNA Sequence Analysis: Methods in Molecular Biology Series Vol. 537. Humana Press, NJ, USA;pp. 337–350 (2009). 10.1007/978-1-59745-251-9_17 19378153

[81] Krzywinski M (2009). Circos: an information aesthetic for comparative genomics. Genome Res..

[82] Conesa A, Gotz S (2008). Blast2GO: a comprehensive suite for functional analysis in plant genomics. Int. J. Plant Genomics..

[83] Szklarczyk D (2011). The STRING database in 2011: functional interaction networks of proteins, globally integrated and scored. Nucl. Acids Res..

[84] Berman HM (2000). The protein data bank. Nucleic Acids Res..

[85] Kelley LA, Sternberg MJE (2009). Protein structure prediction on the Web: a case study using the Phyre server. Nature Protocols.

[86] EbrahimiKhaksefidi R (2015). Differential expression of seven conserved microRNAs in response to abiotic stress and their regulatory network in *Helianthus annuus*. Front. Plant Sci..

[87] Catsky J (1960). Determination of water deficit in disks cut out from leaf blades. Biol. Plant..

[88] Di Caterina R, Giuliani M, Rotunno T, De Caro A, &Flagella Z (2007). Influence of salt stress on seed yield and oil quality of two sunflower hybrids. Ann. Appl. Biol..

[89] Ramu VS (2016). Transcriptome analysis of sunflower genotypes with contrasting oxidative stress tolerance reveals individual- and combined- biotic and abiotic stress tolerance mechanisms. PLoS ONE..

[90] Bates LS, Waldren RP, Teare ID (1973). Rapid determination of free proline for water-stress studies. Plant Soil..

[91] Lata C, Sahu PP, Prasad M (2010). Comparative transcriptome analysis of differentially expressed genes in foxtail millet (*Setaria italica* L.) during dehydration stress. Biochem. Biophys. Res. Commun..

[92] Lata C, Bhutty S, Bahadur RP, Majee M, Prasad M (2011). Association of a SNP in a novel DREB2-like gene SiDREB2 with stress tolerance in foxtail millet (*Setaria italica* L.). J. Exp. Bot..

[93] Kumar K, Muthamilarasan M, Prasad M (2013). Reference genes for quantitative Real-time PCR analysis in the model plant foxtail millet (*Setaria italica* L.) subjected to abiotic stress conditions. Plant Cell Tiss. Organ Cult..

[94] Chen L (2012). Genome-wide identification and analysis of MAPK and MAPKK gene families in *B. distachyon*. PloSOne.

