# Supplementary material for: Characterization of the APETALA2/Ethylene-responsive factor (AP2/ERF) transcription factor family in sunflower
Source: Sci Rep. 2018 Aug 1;8:11576. doi: 10.1038/s41598-018-29526-z (PMC6070487; doi:10.1038/s41598-018-29526-z)
Supplement: Supplementary file 2 — Supporting Information Files [file 41598_2018_29526_MOESM2_ESM.pdf]

Supporting information files

Title of article: Characterization of the APETALA2/Ethylene-responsive factor (AP2/ERF) transcription factor family in sunflower

Somayeh Najafi[1], Karim Sorkheh[1*], Fatemeh Nasernakhaei[1]

[1]Department of Agronomy and Plant Breeding, Faculty of Agriculture, Shahid Chamran University of Ahvaz, P. O. Box 61355/144, Iran

Journal: Scientific Reports- Nature Publishing Groups

[*]**Corresponding author,** Department of Agronomy and Plant Breeding, Faculty of Agriculture, Shahid Chamran University of Ahvaz, P.O. Box 61355/144, Iran. E-mail address: karimsorkheh@gmail.com (K. Sorkheh).

Supporting Information of Figures and Tables

Supporting Information of Figure S

Fig. S1. Highly conserved motif indifferent subfamily AP2/ERF in sunflower

Fig. S2. Gene structures of 288*HaAP2/ERF* proteins. Exons and introns are represented by green boxes and black lines, respectively

Fig. S3. Proline contents in sunflower under cold, salt, drought, and heat (for details of treatment see the methods section) stress

Fig. S4. Na^+/K^+ ratio in leaf and root of sunflower under different salt stress levels

Fig. S5. Effect of different drought levels (control, 12h, 24h, and 48h) on relative water content (RWC) sunflower

Supporting information of Table S

Table S1. Characteristic features of AP2/ERF Transcription factor gene family identified in *Helianthus annuus*

Table S2. Summary of functional domains present in the *HaAP2/ERF* proteins

Table S3. The Ka/Ks ratios and estimated divergence time for tandemly duplicated *HaAP2/ERF* genes.

Table S4. The Ka/Ks ratios and estimated divergence time for segmentally duplicated *HaAP2/ERF* genes

Table S5. The Ka/Ks ratios and estimated divergence time for orthologous *HaAP2/ERF* proteins between *Helianthus annuus* and *Arabidopsis thaliana*

Table S6. The Ka/Ks ratios and estimated divergence time for orthologous *HaAP2/ERF* proteins between *Helianthus annuus* and *Oryza sativa*

Table S7. The Ka/Ks ratios and estimated divergence time for orthologous *HaAP2/ERF* proteins between *Helianthus annuus* and *Glycine max*

Table S8. Summary of abiotic-stress inducible *cis*-elements is in the promoter regions of DREB subfamily genes in *Helianthus annuus*

Table S9. Annotations of *HaDREBs* cis-acting regulatory DNA elements identified in *Helianthus annuus* by PLACE.

Table S10. Interaction network of 9 *HaDREB* genes identified in sunflower and related genes in *Arabidopsis*

Table S11. Forward and reverse primers used in the qRT-PCR genes expression studies
