# Supplementary material for: Characterization of the APETALA2/Ethylene-responsive factor (AP2/ERF) transcription factor family in sunflower
Source: Sci Rep. 2018 Aug 1;8:11576. doi: 10.1038/s41598-018-29526-z (PMC6070487; doi:10.1038/s41598-018-29526-z)
Supplement: Supplementary file 1 — Supplementary Figures [file 41598_2018_29526_MOESM1_ESM.pdf]

# Legends for supplementary of Table S

**Table S1.** Characteristic features of AP2/ERF Transcription factor gene family identified in *Helianthus annuus*

**Table S2.** Summary of functional domains present in the *HaAP2/ERF* proteins

**Table S3.** The Ka/Ks ratios and estimated divergence time for tandemly duplicated *HaAP2/ERF* genes.

**Table S4.** The Ka/Ks ratios and estimated divergence time for segmentally duplicated *HaAP2/ERF* genes

**Table S5.** The Ka/Ks ratios and estimated divergence time for orthologous *HaAP2/ERF* proteins between *Helianthus annuus* and *Arabidopsis thaliana*

**Table S6.** The Ka/Ks ratios and estimated divergence time for orthologous *HaAP2/ERF* proteins between *Helianthus annuus* and *Oryza sativa*

**Table S7.** The Ka/Ks ratios and estimated divergence time for orthologous *HaAP2/ERF* proteins between *Helianthus annuus* and *Glycine max*

**Table S8.** Summary of abiotic-stress inducible *cis*-elements is in the promoter regions of DREB subfamily genes in *Helianthus annuus*

**Table S9.** Annotations of *HaDREBs* cis-acting regulatory DNA elements identified in *Helianthus annuus* by PLACE.

**Table S10.** Interaction network of 9 *HaDREB* genes identified in sunflower and related genes in *Arabidopsis*

**Table S11.** Forward and reverse primers used in the qRT-PCR genes expression studies

# Supplementary FigureS1

**Title of article:** **Characterization of the APETALA2/Ethylene-responsive factor (AP2/ERF) transcription factor family in sunflower**

**Somayeh Najafi[1], Karim Sorkheh[1*], Fatemeh Nasernakhaei[1]**

[1]Department of Agronomy and Plant Breeding, Faculty of Agriculture, Shahid Chamran University of Ahvaz, P. O. Box 61355/144, Iran

**Journal: Scientific Reports- Nature Publishing Groups**

[*]**Corresponding author,** Department of Agronomy and Plant Breeding, Faculty of Agriculture, Shahid Chamran University of Ahvaz, P.O. Box 61355/144, Iran. E-mail address: karimsorkheh@gmail.com (K. Sorkheh).

**Fig. S1.**Highly conserved motif indifferent subfamily AP2/ERF in sunflower

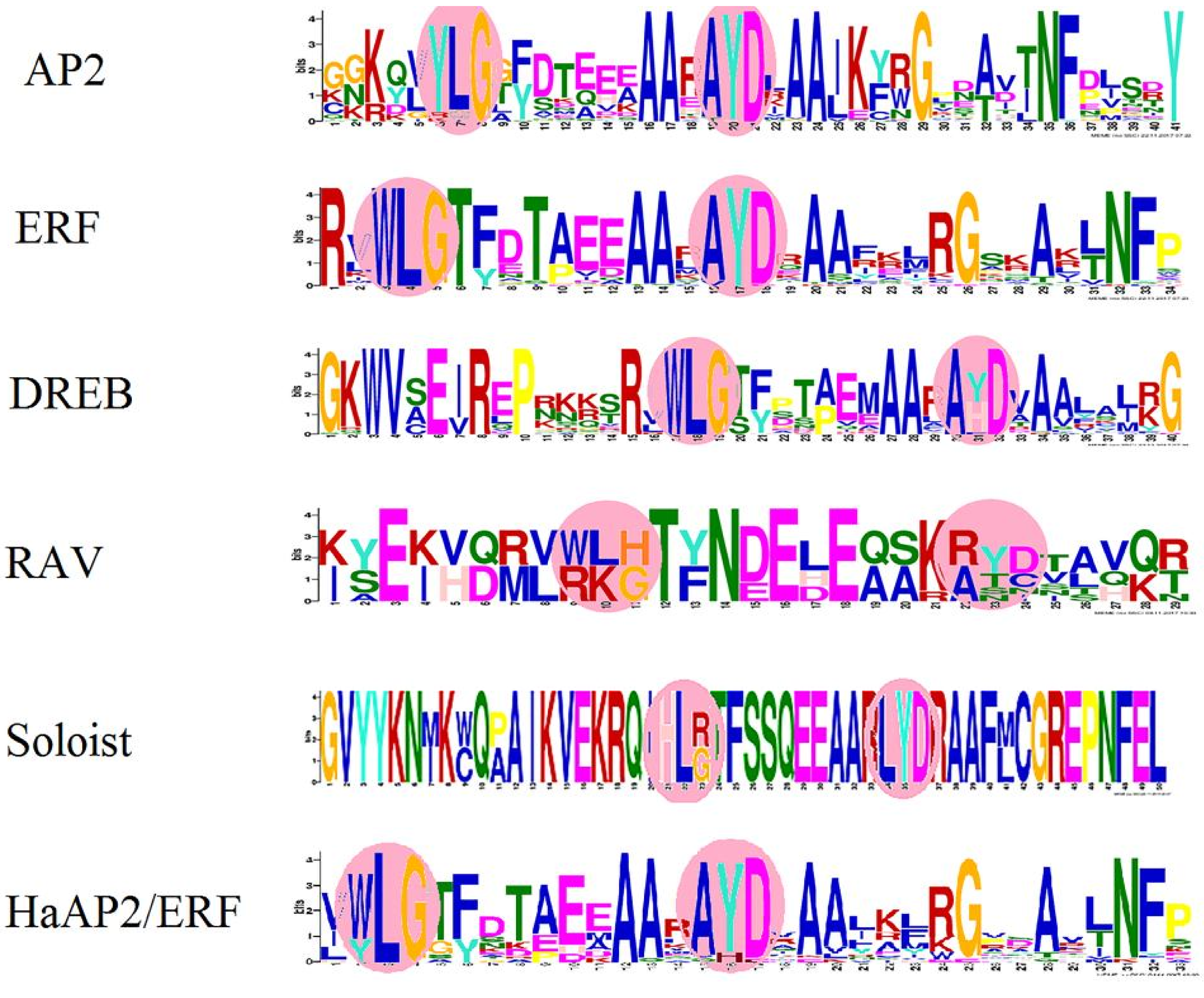
AP2
ERF
DREB
RAV
Soloist
HaAP2/ERF

# Supplementary FigureS2

**Title of article: Characterization of the APETALA2/Ethylene-responsive factor (AP2/ERF) transcription factor family in sunflower**

**Somayeh Najafi[1], Karim Sorkheh[1*], Fatemeh Nasernakhaei[1]**

[1]Department of Agronomy and Plant Breeding, Faculty of Agriculture, Shahid Chamran University of Ahvaz, P. O. Box 61355/144, Iran

**Journal: Scientific Reports- Nature Publishing Groups**

[*]**Corresponding author,** Department of Agronomy and Plant Breeding, Faculty of Agriculture, Shahid Chamran University of Ahvaz, P.O. Box 61355/144, Iran. E-mail address: karimsorkheh@gmail.com (K. Sorkheh).

**Fig. S2.** Gene structures of 288*HaAP2/ERF* proteins. Exons and introns are represented by green boxes and black lines, respectively

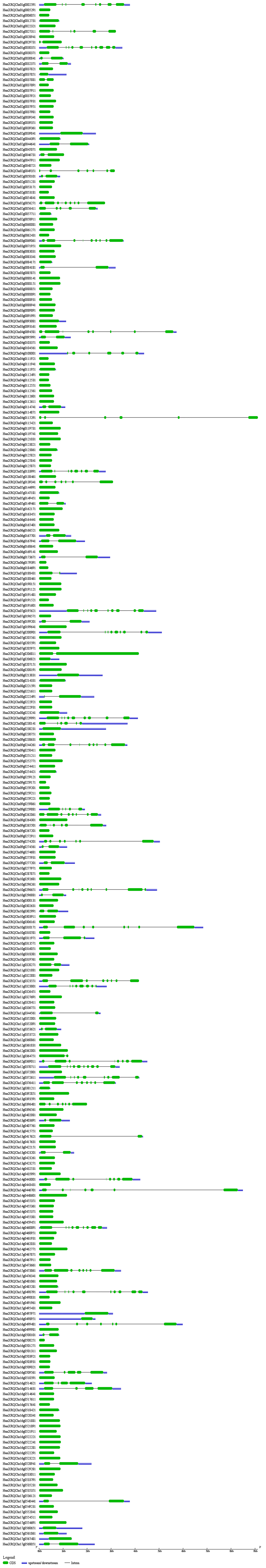
Legend
CDS
upstream/downstream
Intron

# Supplementary FigureS3

**Title of article:** **Characterization of the APETALA2/Ethylene-responsive factor (AP2/ERF) transcription factor family in sunflower**

**Somayeh Najafi[1], Karim Sorkheh[1*], Fatemeh Nasernakhaei[1]**

[1]Department of Agronomy and Plant Breeding, Faculty of Agriculture, Shahid Chamran University of Ahvaz, P. O. Box 61355/144, Iran

**Journal: Scientific Reports- Nature Publishing Groups**

[*]**Corresponding author,** Department of Agronomy and Plant Breeding, Faculty of Agriculture, Shahid Chamran University of Ahvaz, P.O. Box 61355/144, Iran. E-mail address: karimsorkheh@gmail.com (K. Sorkheh).

**Fig. S3.** Proline contents in sunflower under cold, salt, drought, and heat (for details of treatment see the methods section) stress

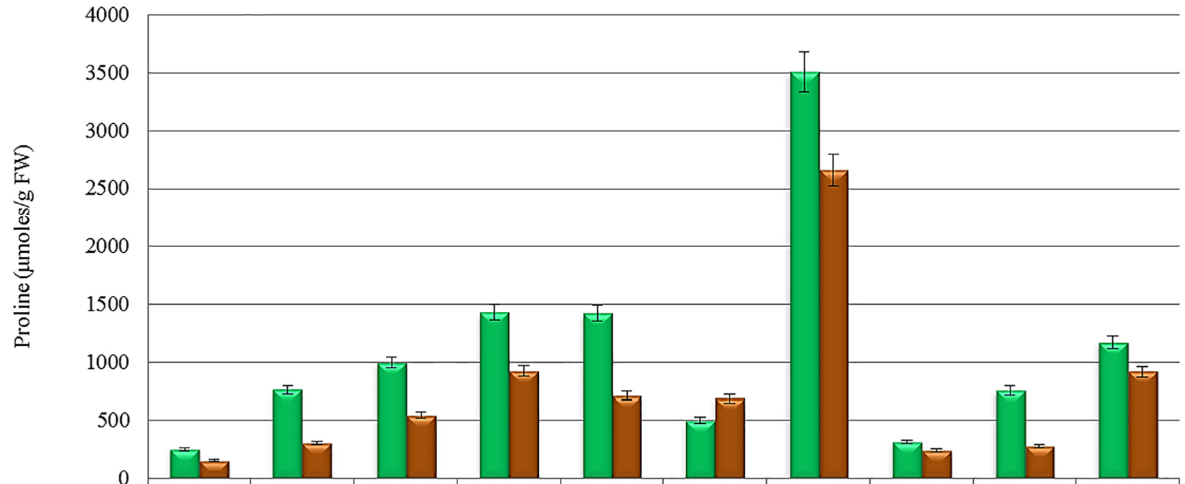


| | Control | Cold | Salt 1 | Salt 2 | Drought 1 | Drought 2 | Drought 3 | Heat 1 | Heat 2 | Heat 3 |
|---|---|---|---|---|---|---|---|---|---|---|
| Proline - Leaf | 254.79283 | 761.90476 | 996.90785 | 1429.8083 | 1423.624 | 502.1645 | 3507.7304 | 316.63574 | 755.72047 | 1170.068 |
| Proline - Root | 155.84416 | 304.26716 | 545.45454 | 922.69635 | 712.43043 | 687.69326 | 2660.4824 | 242.42424 | 279.52999 | 916.51206 |

# Supplementary FigureS4

**Title of article: Characterization of the APETALA2/Ethylene-responsive factor (AP2/ERF) transcription factor family in sunflower**

**Somayeh Najafi[1], Karim Sorkheh[1*], Fatemeh Nasernakhaei[1]**

[1]Department of Agronomy and Plant Breeding, Faculty of Agriculture, Shahid Chamran University of Ahvaz, P. O. Box 61355/144, Iran

**Journal: Scientific Reports- Nature Publishing Groups**

[*]**Corresponding author,** Department of Agronomy and Plant Breeding, Faculty of Agriculture, Shahid Chamran University of Ahvaz, P.O. Box 61355/144, Iran. E-mail address: karimsorkheh@gmail.com (K. Sorkheh).

**Fig. S4.** $Na^+/K^+$ ratio in leaf and root of sunflower under different salt stress levels

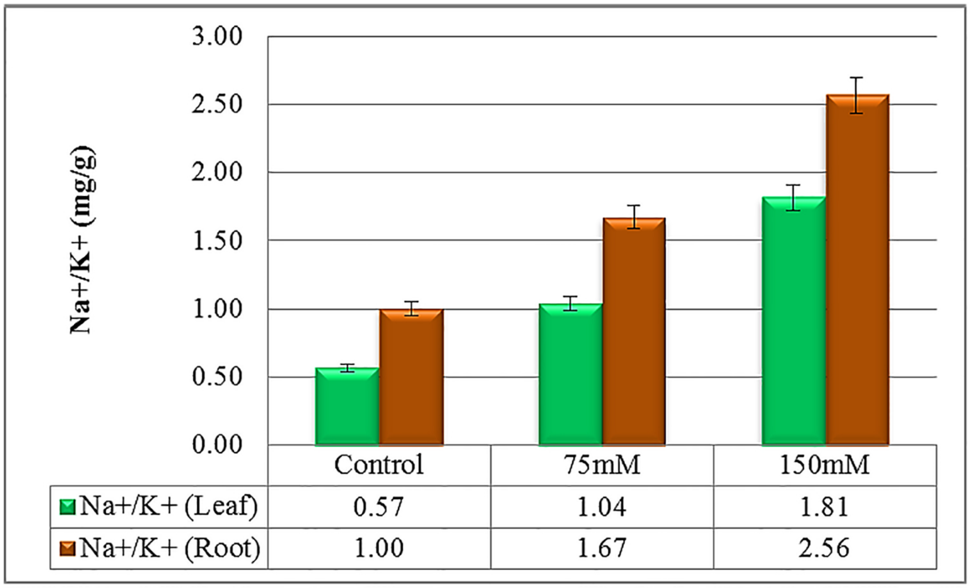
Na+/K+ (mg/g)
3.00
2.50
2.00
1.50
1.00
0.50
0.00
Control
75mM
150mM
Na+/K+ (Leaf) 0.57 1.04 1.81
Na+/K+ (Root) 1.00 1.67 2.56

# Supplementary FigureS5

**Title of article: Characterization of the APETALA2/Ethylene-responsive factor (AP2/ERF) transcription factor family in sunflower**

**Somayeh Najafi[1], Karim Sorkheh[1*], Fatemeh Nasernakhaei[1]**

[1]Department of Agronomy and Plant Breeding, Faculty of Agriculture, Shahid Chamran University of Ahvaz, P. O. Box 61355/144, Iran

**Journal: Scientific Reports- Nature Publishing Groups**

[*]**Corresponding author,** Department of Agronomy and Plant Breeding, Faculty of Agriculture, Shahid Chamran University of Ahvaz, P.O. Box 61355/144, Iran. E-mail address: karimsorkheh@gmail.com (K. Sorkheh).

**Fig. S5.** Effect of different drought levels (control, 12h, 24h, and 48h) on relative water content (RWC) sunflower

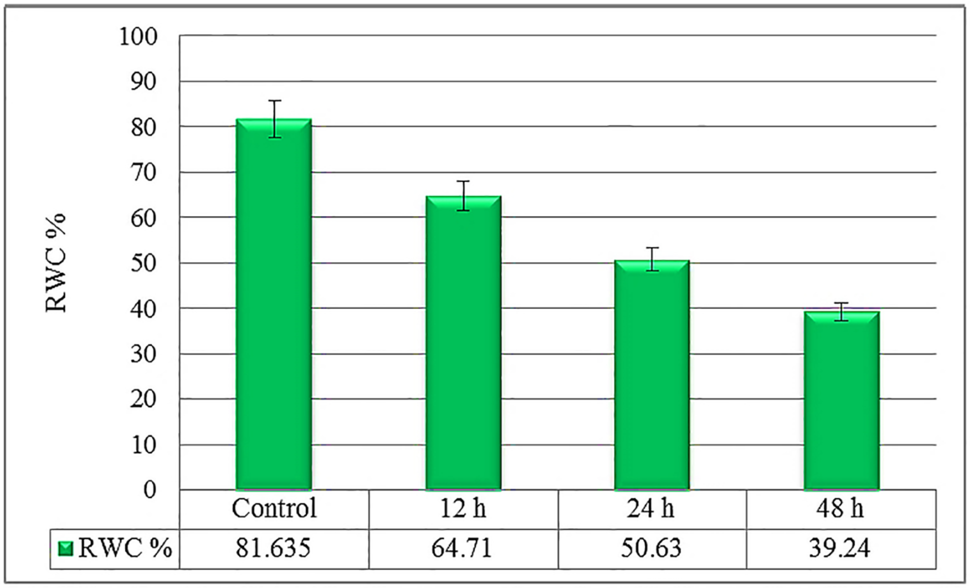
100
90
80
70
60
50
40
30
20
10
0
RWC %
Control
12 h
24 h
48 h
RWC %
81.635
64.71
50.63
39.24
